# Genome-wide transcriptional changes triggered by water deficit on a drought-tolerant common bean cultivar

**DOI:** 10.1186/s12870-020-02664-1

**Published:** 2020-11-17

**Authors:** Josefat Gregorio Jorge, Miguel Angel Villalobos-López, Karen Lizeth Chavarría-Alvarado, Selma Ríos-Meléndez, Melina López-Meyer, Analilia Arroyo-Becerra

**Affiliations:** 1 Consejo Nacional de Ciencia y Tecnología - Centro de Investigación en Biotecnología Aplicada, Instituto Politécnico Nacional (CIBA-IPN), Ex-Hacienda San Juan Molino, Carretera Estatal Tecuexcomac- Tepetitla de Lardizábal Km 1.5, 90700 Tlaxcala, Mexico; 2 Laboratorio de Genómica Funcional y Biotecnología de Plantas, Centro de Investigación en Biotecnología Aplicada, Instituto Politécnico Nacional (CIBA-IPN), Ex-Hacienda San Juan Molino, Carretera Estatal Tecuexcomac- Tepetitla de Lardizábal Km 1.5, 90700 Tlaxcala, Mexico; 3 Departamento de Biotecnología Agrícola, Centro Interdisciplinario de Investigación para el Desarrollo Integral Regional, Instituto Politécnico Nacional (CIIDIR-IPN Unidad Sinaloa), Boulevard Juan de Dios Bátiz Paredes 250, Colonia San Joachin, 81101 Guasave, Sinaloa Mexico

**Keywords:** Common bean, *P. vulgaris*, Drought, Abiotic stress, Cell wall, RNA-seq

## Abstract

**Background:**

Common bean (*Phaseolus vulgaris L.*) is a relevant crop cultivated over the world, largely in water insufficiency vulnerable areas. Since drought is the main environmental factor restraining worldwide crop production, efforts have been invested to amend drought tolerance in commercial common bean varieties. However, scarce molecular data are available for those cultivars of *P. vulgaris* with drought tolerance attributes.

**Results:**

As a first approach, Pinto Saltillo (PS), Azufrado Higuera (AH), and Negro Jamapa Plus (NP) were assessed phenotypically and physiologically to determine the outcome in response to drought on these common bean cultivars. Based on this, a Next-generation sequencing approach was applied to PS, which was the most drought-tolerant cultivar to determine the molecular changes at the transcriptional level. The RNA-Seq analysis revealed that numerous PS genes are dynamically modulated by drought. In brief, 1005 differentially expressed genes (DEGs) were identified, from which 645 genes were up-regulated by drought stress, whereas 360 genes were down-regulated. Further analysis showed that the enriched categories of the up-regulated genes in response to drought fit to processes related to carbohydrate metabolism (polysaccharide metabolic processes), particularly genes encoding proteins located within the cell periphery (cell wall dynamics). In the case of down-regulated genes, heat shock-responsive genes, mainly associated with protein folding, chloroplast, and oxidation-reduction processes were identified.

**Conclusions:**

Our findings suggest that secondary cell wall (SCW) properties contribute to *P. vulgaris* L. drought tolerance through alleviation or mitigation of drought-induced osmotic disturbances, making cultivars more adaptable to such stress. Altogether, the knowledge derived from this study is significant for a forthcoming understanding of the molecular mechanisms involved in drought tolerance on common bean, especially for drought-tolerant cultivars such as PS.

## Background

Water has become the most significant limiting factor in the world of agriculture, and therefore, affects the welfare of the human population. The increase in population around the world is driving up a huge demand for food, accompanied by the intensification of deforestation to create new farmland areas. More than a third of the earth’s surface consists of arid and semi-arid zones characterized by low rainfall that parallels low productivity in plants. This situation worsens due to global warming that has caused climate changes, which has negative impacts on agronomic activities that threaten food security [1–3]. Whereas climate changes have intensified precipitation in some areas, in other regions it has contributed to rainless and aridity. In México, the distribution of water resources is a worrying problem, since more than half of the country has desert and semi-desert characteristics. In addition, high temperatures and rainfall insufficiency have increased arid areas [4]. Therefore, numerous regions, where drought is already a challenge, will suffer from warmer and drier weather over the next few decades [5–8]. Thus, it is not surprising that drought is considered one of the major and most catastrophic environmental factors that negatively affect plant productivity and survival around the world [9–11].

Plants, being sessile organisms, have developed sophisticated mechanisms to confront environmental challenges [12, 13]. Although the damage caused by drought in plants depends on its extent and intensity, it affects overall plant growth by altering critical biological processes such as photosynthesis and nutrient assimilation [14, 15]. To cope with drought spells, plants trigger diverse phytohormone signaling, antioxidant and metabolite production and mobilization systems, in order to activate tissue water retention, osmotic adjustment, integrity of membrane system, and stomata adjustment, increase root water uptake, among others to maintain physiological water balance [16, 17]. In the case of common bean (*Phaseolus vulgaris* L.), a Mesoamerican originated legume crop that represents an essential plant protein source in developing countries such as those of Latin America and Africa, is relatively sensitive to drought stress compared to other legumes [18]. Although drought affects common bean growth and development at every stage of its life cycle, most of the studies have focused on vegetative and reproductive stages, being seed yield as the primary trait measured [16]. On the other hand, cultivated common bean varieties are classified into two well-defined genetic pools (Middle American and Andean), which are subdivided in landraces [19–23]. Despite *P. vulgaris* importance and its genetic diversity, with approximately 2900 records of cultivated varieties [24], the genomic information sources of common beans are limited. Until recently, remarkable efforts have been made to generate collections of *P. vulgaris* L. sequences [25–33]. In México, varieties belonging to the Middle American (Durango, Jalisco, and Mesoamerica) and Andean (Nueva Granada) genetic landraces are cultivated.

Considering the common bean sensitivity to drought stress, the improvement of drought tolerance has been one of the primary goals of breeding programs of this important crop [18, 34, 35]. Wild beans have been excellent genetic sources to improve currently used common bean cultivars, especially wild beans from semiarid regions of México [36–38]. Those efforts have derived into the development of cultivars tolerant to drought, such as Pinto Saltillo (PS), a commercial cultivar that is a member of the Durango race [35, 39]. Although the Durango race is the only group that contains cultivars with significant drought tolerance [23], other cultivars have been successfully cultivated in the north of México. Among such cultivars is the black bean landrace known as Negro Jamapa 81, which has been the most studied Mesoamerican cultivar at the molecular level [31, 40–43]. Another high yield bean cultivar is Azufrado Higuera (AH), belonging to the Nueva Granada race, which is the most widely cultivated Andean race in the north of México [44, 45].

According to the Agency of marketing services and development of agricultural markets of Mexico (ASERCA), it has been estimated that PS, Negro Jamapa, and AH represent around 70% of the national bean production [46]. Thus, a comparison among these common bean genotypes concerning drought-derived effects is scarce and necessary. Moreover, since withstanding water deficit during the vegetative phase of *P. vulgaris* determines good yields under drought conditions, here we analyzed physiological parameters of PS, Negro Jamapa Plus (NP), a purified version of Negro Jamapa 81, and AH common bean cultivars under drought stress. Based on this analysis, a genome-wide approach was applied to the most drought-tolerant cultivar, namely the RNA profiling of PS after 2 weeks of drought. Taken together, the assessment of drought tolerance of PS at the physiological and molecular level shed light into the putative molecular mechanisms of how this common bean cultivar responds and adapts to drought.

## Results

### Differential response of three common bean cultivars subjected to drought stress

**Fig. 1:**
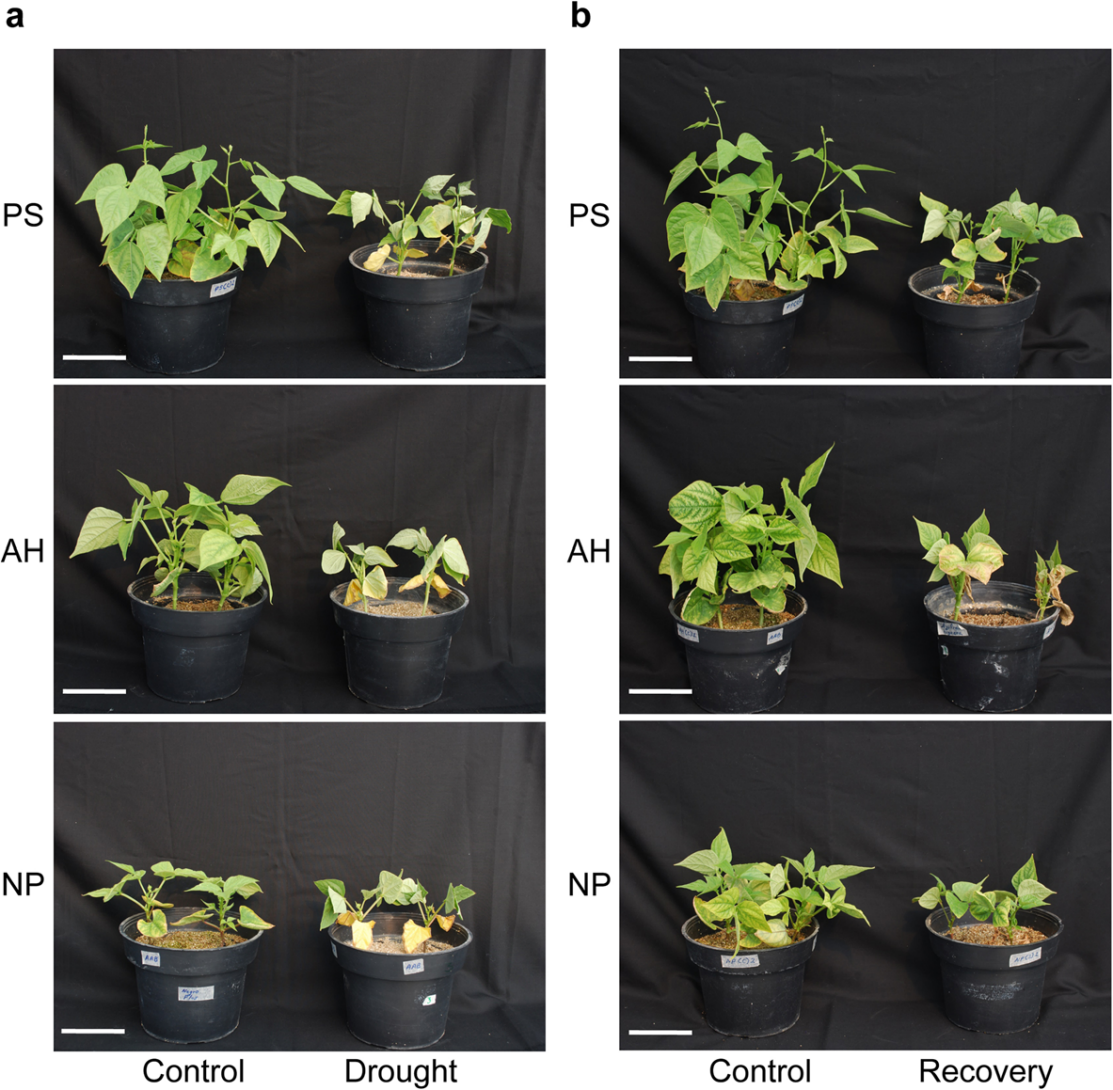
Effect of drought stress on the phenotypic appearance of three common bean cultivars. **a.** Phenotypic appearance of bean cultivars after two weeks of drought stress. **b.** Phenotypes of bean cultivars after two weeks of recovery. Pictures are representative of at least three independent experiments. Pinto Saltillo (PS), Azufrado Higuera (AH), and Negro Jamapa Plus (NP). Scale bar = 10 cm

**Fig. 2:**
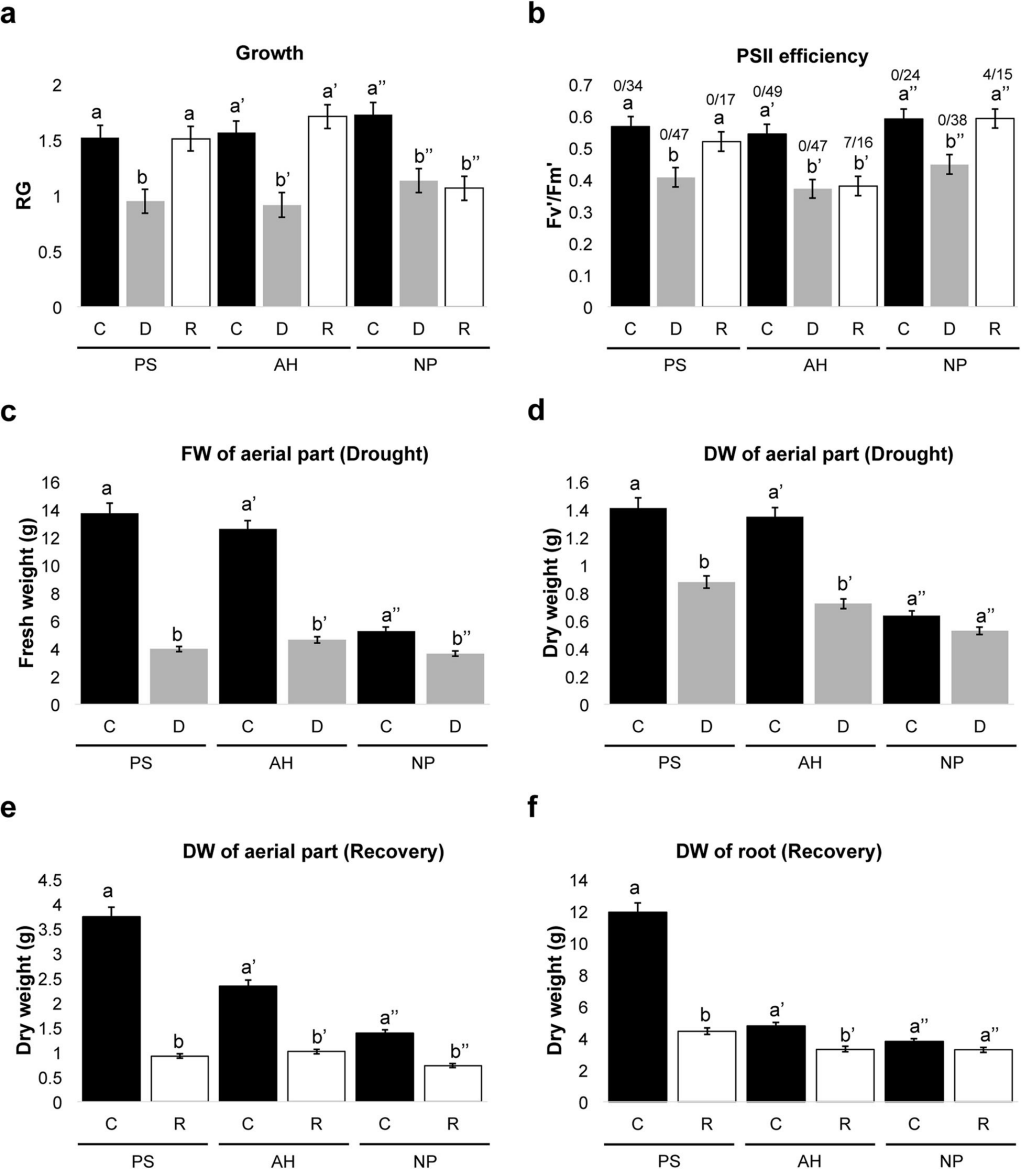
Changes in physiological parameters of three common bean cultivars in response to drought. **a.** Relative growth (RG) values of bean cultivars after two weeks of drought stress (sixty-days after transplanting), as well as RG values after two weeks of re-hydration (seventy-four-days after transplanting). **b.** Values of PSII efficiency (*F*_*v*_*’/F*_*m*_*′*) of bean cultivars at the end of drought treatment, and after two weeks of recovery (re-watering) are shown for the first three trifoliates. Numbers above bars indicate the number of senescent leaves in each case. **c** and **d** Fresh weight (FW) and Dry weight (DW) of the aerial parts of well-watered and drought-stressed plants (sixty-days after transplanting), respectively. **e** and **f** DW of the aerial and root parts of control and re-watered plants (seventy-four-days after transplanting), respectively. Pinto Saltillo (PS), Azufrado Higuera (AH), and Negro Jamapa Plus (NP). C, Control; D, Drought; R, Recovery. Graphical representation of mean ± SE of six to nine individual plants from each experiment, out of at least two independent biological experiments. One-way ANOVA was used to compare the statistical difference between measurements (*P* < 0.05). Different letters indicate significant differences compared to the control plants

Common bean plants were subjected to a period of progressive water deficit for 2 weeks by suppression of watering. In contrast, control plants were watered all the time. After 2 weeks of water withdrawal, all common bean plants showed clear symptoms of drought (Fig. 1a). Regular irrigation of all drought-treated plants was re-established to determine whether these common bean cultivars could recover after the drought treatment. Two weeks later, post-drought recovery was assessed (Fig. 1b). Relative growth (RG) values showed that all bean cultivars indeed slowed their growth after 2 weeks of drought stress (Fig. 2a). In the case of photosystem II (PSII) efficiency, as measured by the Quantum yield (equivalent to *F*_*v*_*’/F*_*m*_*′*, ratio of variable to maximum fluorescence of open PSII in light-pre-adapted plants), a reduction was observed in all three varieties (Fig. 2b). The reduction of the PSII efficiency was only true for trifoliates and not for the first true leaves (Additional file 1: Fig. S1). The negative effect on RG was observed since 1 week of drought treatment when compared to the control condition of the same age, where it was observed that the three cultivars stopped their growth capacity (Additional file 2: Fig. S2a). On the other hand, the *F*_*v*_*’/F*_*m*_*′* parameter was sensitive to the water deficit, since the PSII efficiency decreased after 1 week of drought treatment in the three cultivars, and this decrease was accentuated at 14 days of drought. (Additional file 2: Fig. S2b). Although the reduction of growth, as well as the PSII efficiency, followed a similar fashion, determination of the fresh and dry weight of plants after 2 weeks of drought showed a remarkable difference among varieties (Fig. 2c and d). PS and AH exhibited the highest FW and DW compared to NP (Fig. 2c and d); however, PS showed the highest DW values of the aerial part after drought stress (Fig. 2d). Although a correlation was observed between FW and DW values in the case of well-watered control plants, in which DW values were 10 % of those of FW, PS cultivar exhibited the major difference between FW and DW values under the drought treatment (Additional file 3: Fig. S3). On the other hand, a look into the RG values after recovery showed that PS and AH cultivars increased their growth, whereas NP did not, evidencing the capacity of PS and AH to re-start growth after drought stress (Fig. 2a). In the case of the PSII efficiency in recovery conditions, only PS and NP trifoliates were capable of recovering PSII efficiency, and AH was not (Fig. 2b). A striking observation is that PS plants, on which the PSII efficiency was measured, did not present senescent leaves after 2 weeks of re-watering, whereas AH and NP showed senescent leaves (Fig. 2b). Finally, DW values of the aerial and root parts of plants belonging to the group of the post-drought recovery assay (72 days-old) showed that control plants of PS had remarkable higher biomass in comparison to AH and NP (Fig. 2e and f, and Additional file 3: Fig. S3b). In summary, the measurements of physiological features of three common bean cultivars subjected to drought stress and then re-watered for recovery, indicate that during drought stress PS suffered less damage in leaves, had the highest DW values of aerial part, and had the highest FW and DW under control conditions. In addition, in the post-drought recovery assay, PS appearance was not wilty, greener leaves, more robust, showed a good capacity to re-start growth, recovered normal PSII efficiency and had high root DW values; leading to conclude that PS cultivar has better drought tolerance capacities than AH and NP varieties, although the latter also have good traits under water deficit conditions.

### RNA profiling of PS after two weeks of drought stress

**Fig. 3:**
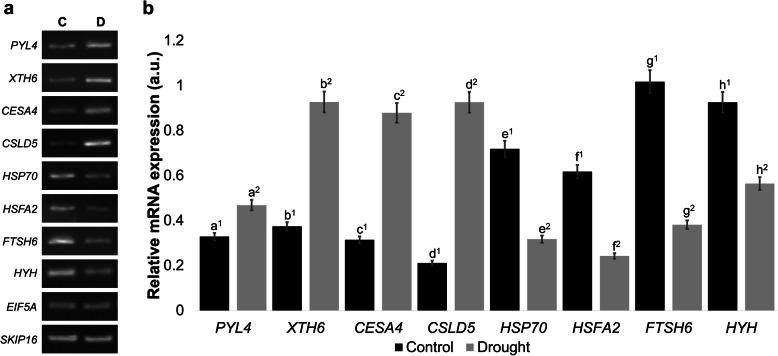
Validation of selected DEGs determined by semi-quantitative RT-PCR. a. RT-PCR analysis by agarose gel electrophoresis of up- (*PYL4, XTH6*, *CESA4*, and *CSLD5*) and down-regulated (*HSP70*, *HSFA2*, *FTSH6*, and *HYH*) genes are shown for PS. Constitutive genes from our RNA-Seq data (*EIF5A*) and previously reported (*SKIP16*) were used in the analysis. Representative gels corresponding to 32 (*CESA4, CSLD5,* and *HSP70*) and 34 (*PYL4, XTH6, HSFA2, FTSH6, HYH, EIF5A,* and *SKIP16*) cycles are shown. (C, Control; D, Drought). **b.** Density analysis of PCR bands was determined by ImageJ software and normalized using the *EIF5A* constitutive internal control corresponding to each condition (a.u. - arbitrary units). Graphical representation of mean ± SE of at least three independent replicates. One-way ANOVA was used to compare the statistical difference between measurements (*P* < 0.05). Samples tested for the same gene are indicated by lowercase letters. Significant differences compared to the control samples are indicated by different numbers

Since PS has previously been described as a drought-tolerant cultivar [35, 39, 47], and showed better tolerance to drought than AH and NP, such cultivar was assessed to get insights into the molecular mechanisms that could contribute to its tolerance, as a first approach, the transcriptome of aerial tissues on this common bean cultivar was examined using the RNA-Seq technology. The total number of preprocessed reads, with an average read length of 36 bp, ranged from 51 to 56 million (Table 1). Then, reads were aligned to the *P. vulgaris* reference genome with TopHat/Bowtie, a fast splice junction mapper proper for short reads. A high percentage of uniquely mapped reads were obtained, whereas reads that did not map were low (Table 1). In the case of the control condition, 91.7% of the reads were uniquely mapped in the genome, whereas 93.5% were mapped in the drought condition (Table 1). Expression levels of genes were determined using Cuffdiff, taking into account the FPKM values (Additional file 4: Fig. S4). Overall, 1005 putative differentially expressed genes (DEGs) were identified, from which 645 genes were found to be up-regulated by the drought treatment, whereas 360 were down-regulated (Table 1 and Additional file 5: Table S1). Semi-quantitative RT-PCR analyses for some selected DEGs according to the Functional association networks (see below) were performed for validation (Fig. 3 and Additional file 6: Fig. S5). Accordingly, *PYL4, XTH6*, *CESA4,* and *CSLD5,* which were found to be up-regulated in the RNA-Seq data, were confirmed as induced in the RT-PCR analysis (Fig. 3 and Additional file 6: Fig. S5). On the other hand, the expression of *HSP70*, *HSFA2*, *FTSH6,* and *HYH*, which were down-regulated genes in the dataset, were reduced in the drought stress condition as assessed by RT-PCR (Fig. 3 and Additional file 6: Fig. S5). Some of these DEGs were also tested in the other two cultivars of common bean, namely AH and NP, showing a similar response mainly for up-regulated genes (Additional file 7: Fig. S6). As RNA samples for semi-quantitative RT-PCR assays were different from those used for RNAseq, but from independent experiments under the same control and drought stress conditions, this independent verification supports the reproducibility and reliability of our transcriptome analysis, and validates the RNA-seq data. Altogether, the RNA-Seq analysis shows that multiple genes of PS are modulated by drought stress.

**Table 1:** Mapping results of PS RNA-Seq reads

Sample	Preprocessed reads	Uniquely mapped reads (%)	Unmapped (%)	Up-regulated	Down-regulated
Control	56,558,482	51,848,176 (91.7)	4,577,494 (8.8)		
Drought	51,367,879	48,016,093 (93.5)	4,562,461 (9.5)	645	360

### Enrichment analysis of DEGs upon drought stress in PS

**Fig. 4:**
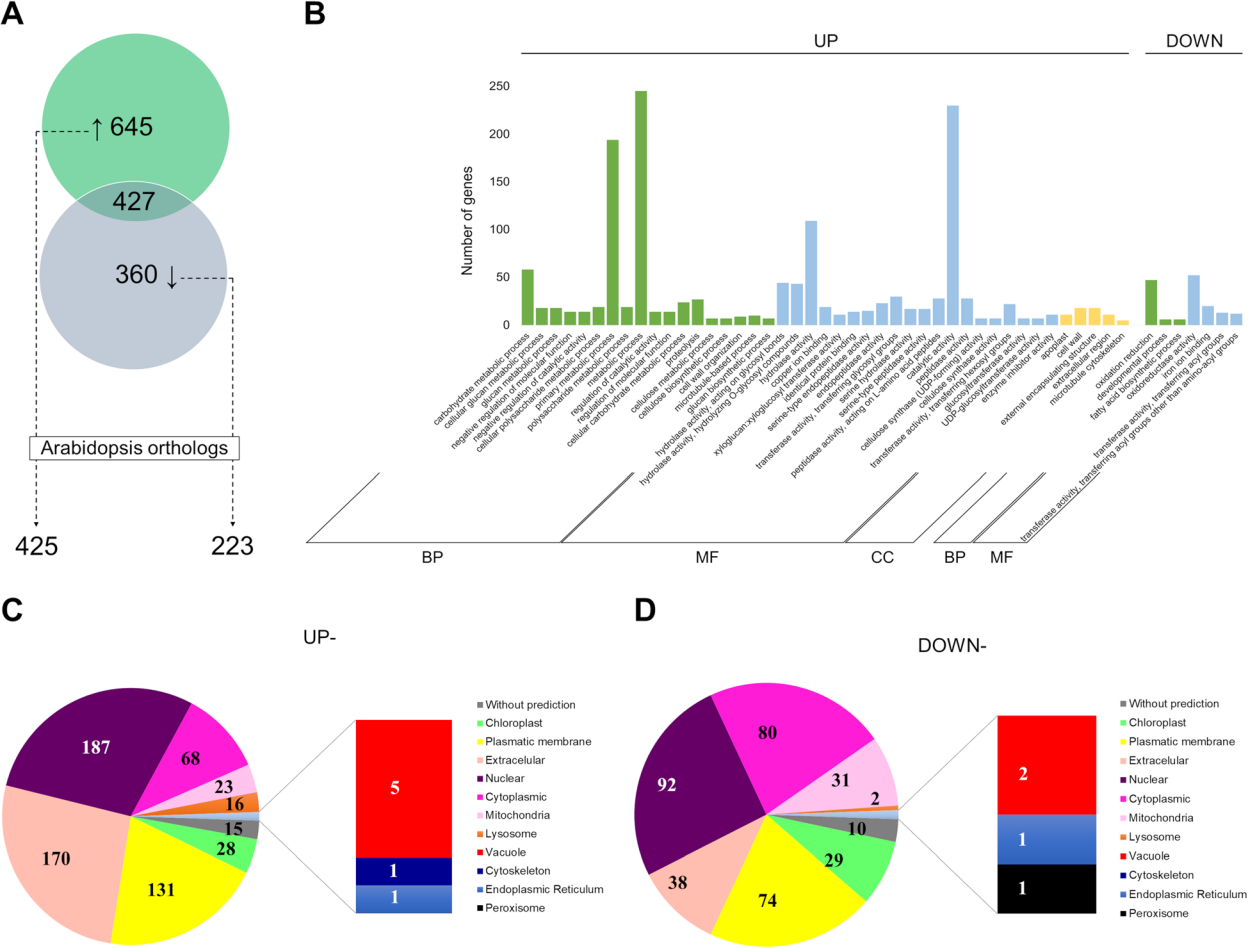
Classification of PS DEGs in response to drought stress. **a**. Venn diagram showing the number of up- and down-regulated genes in response to drought stress. Genes with no expression changes are also shown. The numbers of Arabidopsis orthologs corresponding to the up- and down-regulated genes are shown below the Venn diagram. **b.** Gene ontology (GO) terms enriched or depleted among the up- and down-regulated genes according to Biological process (BP), Molecular function (MF), or Cellular compartment (CC) are shown. **c** and **d** Subcellular classification of up- and down-regulated genes in response to drought stress respectively

Transcriptional changes took place in the PS cultivar in response to drought stress involving numerous up- and down-regulated genes (Fig. 4a and Additional file 5: Table S1). To find out the biological significance of such DEGs during drought, we made a Gene ontology (GO) enrichment analysis of up- and down-regulated genes in relation to Biological process, Molecular function, or Cellular component. The singular enrichment analysis (SEA) performed with the AgriGO tool revealed that significant GO terms were enriched in the set of DEGs (Fig. 4b). Accordingly, 43 GO terms were found enriched in the case of up-regulated genes (Fig. 4b), from which 18 correspond to Biological processes, 20 to Molecular function, and five to Cellular component (Additional file 8: Table S2). On the other hand, down-regulated genes contained only seven GO terms (Fig. 4b). Besides the lower number of GO terms found in the group of down-regulated genes, this set of DEGs did not contain the Cellular component classification but did contain three and four GO terms corresponding to Biological process and Molecular function respectively (Additional file 8: Table S2). Among the first GO terms significantly enriched within the Biological process category corresponding to up-regulated genes, there were processes involved in carbohydrate metabolism, such as carbohydrate metabolic process (58 genes), cellular glucan metabolic process (18 genes) and glucan metabolic process (18 genes) (Fig. 4b and Additional file 8: Table S2). Consistent with this, GO terms corresponding to Molecular function and Cellular component also suggested that most of the up-regulated genes of PS during drought treatment were involved in carbohydrate metabolism in the cell periphery (Fig. 4b and Additional file 8: Table S2). In the case of GO terms found within the down-regulated genes, Biological and Molecular processes identified a tendency to oxidation-reduction/oxidoreductase activity categories (Fig. 4b and Additional file 8: Table S2). The lack of GO terms associated with Cellular component among the down-regulated genes encouraged to predict the subcellular localization of this group of DEGs, as well as of the up-regulated genes. According to the CELLO predictor, up-regulated DEGs had the highest proportion of proteins localized in the cell periphery considering extracellular proteins (170, 26.36%) and plasmatic membrane-associated proteins (131, 20.31%), followed by nuclear-localized predicted proteins (187, 28.99%), cytoplasmic (68, 10.54%), chloroplast (28, 4.34%), mitochondria (23, 3.57%), lysosome (16, 2.48%), vacuole (5, 0.77%), cytoskeleton (1, 0.16%) and endoplasmic reticulum (1, 0.16%); besides 15 proteins without prediction (2.33%) (Fig. 4c). In contrast, down-regulated genes increased the proportions of cytoplasmic (80, 22.22%), mitochondria (31, 8.61%) and chloroplast (29, 8.06%) localized proteins, whereas extracellular proteins (38, 10.56%) decreased (Fig. 4d). Similar proportions of proteins were predicted for subcellular localization in the nucleus (92, 25.56%), plasmatic membrane (74, 20.56%), lysosome (2, 0.56%), vacuole (2, 0.56%), endoplasmic reticulum (1, 0.28%) and proteins without prediction (10, 2.78%) under up- and down-regulated genes; besides one peroxisome protein (0.28%) in down-regulated genes (Fig. 4c and d).

An additional analysis considering only those DEGs with orthologs in Arabidopsis (Fig. 4a) showed the same tendency, namely that up-regulated genes were mainly associated with carbohydrate metabolism in the cell periphery, whereas down-regulated genes were classified as responsive to abiotic stress (Additional file 9: Fig. S7 and Additional file 10: Table S3). Particularly, in the case of up-regulated genes classified within the Biological process category, such DEGs were enriched, among others, in the following GO terms: cell wall organization or biogenesis, polysaccharide metabolic process, polysaccharide biosynthetic process, carbohydrate metabolic process, cell wall macromolecule metabolic process, and glucan metabolic process (Additional file 10: Table S3). In the case of the Cellular component category, this classification showed that up-regulated genes were mainly associated with cell wall-membrane-cytoskeleton continuum (cell periphery), as reflected by the following GO terms: external encapsulating structure, cell wall, extracellular region, intrinsic to the plasma membrane, anchored to the membrane, apoplast, cell-cell junction, and plasmodesma (Additional file 10: Table S3). On the other hand, Arabidopsis orthologs corresponding to down-regulated genes showed enrichment of Biological processes related to abiotic stress response, whereas GO terms associated with Cellular component were depleted (Additional file 9: Fig. S7 and Additional file 10: Table S3). Thus, GO enrichment analysis suggests that most of the up-regulated genes in PS in response to drought belong to processes related to carbohydrate metabolism within the cell periphery, whereas down-regulated genes are associated with an abiotic stress response.

### Representative biological pathways in response to drought stress in PS

To further unraveling possible biological pathways significantly enriched within the up- and down-regulated genes in response to drought stress in the PS cultivar, DEGs with orthologs in Arabidopsis were subjected to analysis using PANTHER. As result, genes involved in Polysaccharide metabolic processes were overrepresented within the up-regulated genes of PS, whereas protein folding was the biological pathway enriched within the down-regulated genes (Additional file 11: Fig. S8). Additional analysis with GENEMANIA and DAVID supported the results obtained by PANTHER (Additional file 12: Table S4).

**Fig. 5:**
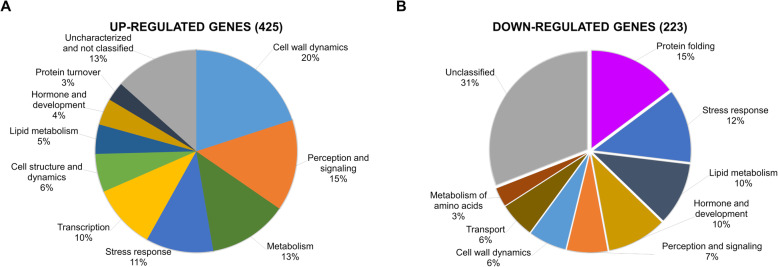
Classification of Arabidopsis orthologs of PS DEGs according to cellular processes. **a** and **b** Pie charts that display clockwise the classification of up- and down-regulated genes of PS corresponding to Arabidopsis orthologs in response to drought stress, respectively

Based on these results, all those Arabidopsis orthologs of DEGs PS genes were grouped according to cellular processes (Fig. 5 and Additional file 13: Table S5). In the case of the 425 orthologs corresponding to up-regulated genes, such DEGs formed 10 groups according to different cellular processes (Fig. 5a and Additional file 13: Table S5). Genes classified into the group of cell wall dynamics were the most prominent (85), followed by perception and signaling (62), metabolism (54), stress response (46), transcription (44), cell structure and dynamics (26), lipid metabolism (20), hormone and development (18), protein turnover (13), as well as unclassified genes (57) (Fig. 5a). On the other hand, among the 223 orthologs for downregulated genes (Additional file 13: Table S5), grouping into different cellular processes resulted in nine groups: protein folding (33), stress response (27), lipid metabolism (23), hormone and development (22), perception and signaling (15), cell wall dynamics (14), transport (13), metabolism of amino acids (7), and unclassified functions (69) (Fig. 5b). Taken together, classification of Arabidopsis orthologs corresponding to PS DEGs showed that the most prominent group of up-regulated genes belong to cell wall dynamics, whereas protein folding is the most remarkable cellular process within the down-regulated genes.

### Functional association networks among DEGs with orthologs in Arabidopsis

**Fig. 6:**
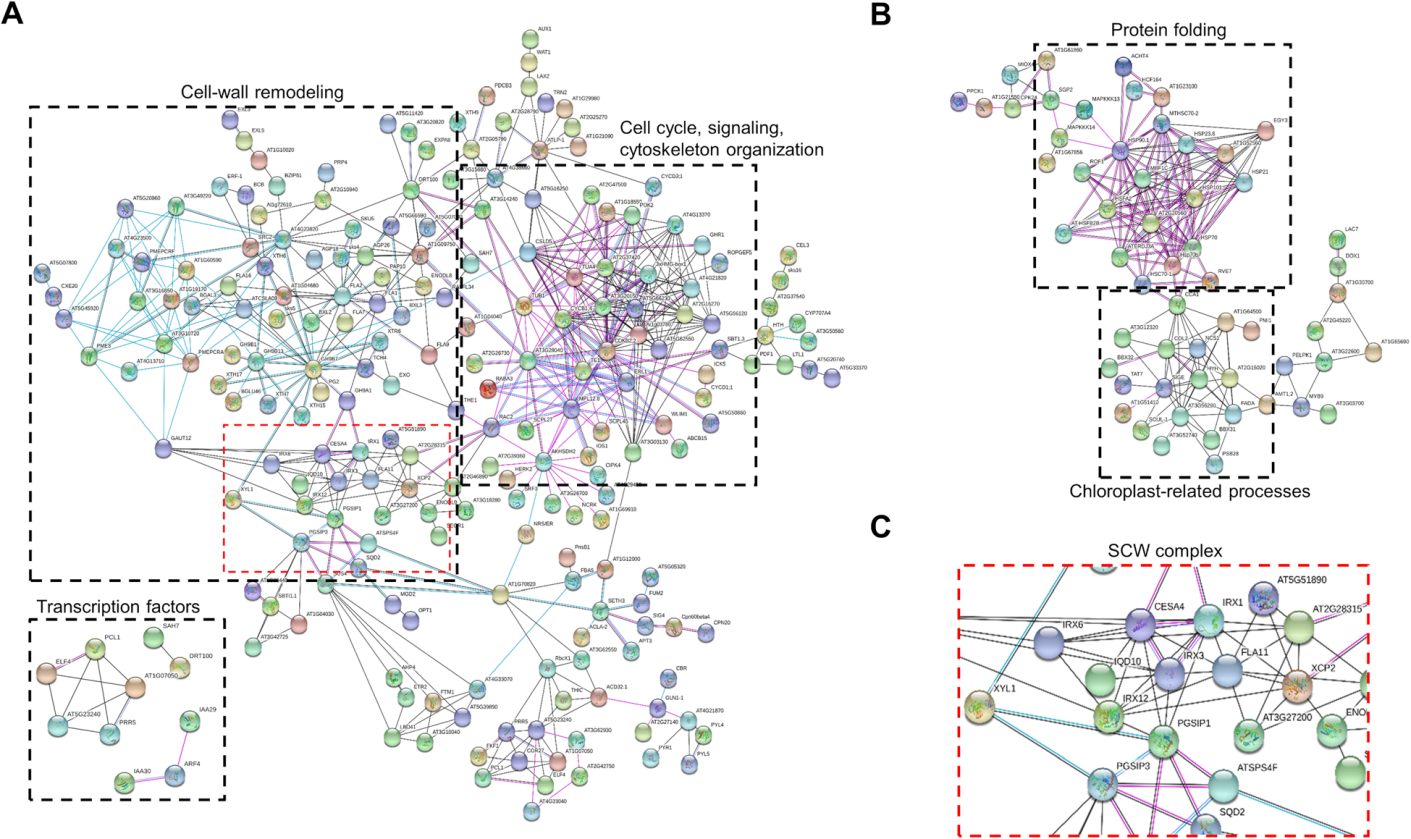
Functional association networks of Arabidopsis orthologs of PS DEGs in response to drought stress. Arabidopsis orthologs forming networks are shown (each node represents a gene). **a** Interactions among the up-regulated genes. **b** Interactions among the down-regulated genes. **c** Subnetwork of the cellulose synthase complex (CSC) from secondary cell wall (SCW). Black dashed rectangles in **a** and **b** indicate subnetworks that protrude from the main network or formed an independent network (transcription factors). Dashed rectangle in red within the subnetwork of cell-wall remodeling indicates components of the CSC from SCW. Colored lines between nodes indicate the various types of interaction evidence: black line, co-expression; light blue line, association in curated databases; purple line, experimental

As gene products do not function in isolation within cells, a network was generated to highlight interactions and relationships between different genes. The orthologs corresponding to the up- and down-regulated genes (Additional file 13: Table S5) were subjected to analysis using the String software to construct an interaction network. Among the seven types of evidence used to predict associations, only three were specified to be displayed: association in curated databases (light blue line), co-expression (black line) and experimental (purple line). As shown in Fig. 6, a large proportion of up- and down-regulated genes have more interactions among themselves than what it would be expected for a random set of proteins of similar size. Specifically, 225 up-regulated genes out of the 425 orthologs interacted with each other, forming identifiable subnetworks (Fig. 6a). A detailed inspection of such subnetworks indicates that they are associated with cell wall remodeling as well as to cell cycle, signaling, or cytoskeleton organization (Fig. 6a). Notably, most of the interactions contained within the subnetworks were of the kind derived from curated databases and co-expression, but also several interactions were supported by experimental data (Fig. 6a and b). Genes located at central nodes were involved in cell wall dynamics, such as *CESA4* (*Cellulose synthase A4*), *IRX1* (*Irregular xylem 1*), *IRX3* (*Irregular xylem 3*), *IRX6* (*Irregular xylem 6*), *IRX12* (*Irregular xylem 12*), *PGSIP1* (*Plant glycogenin-like starch initiation protein 1*) and *PGSIP3* (*Plant glycogenin-like starch initiation protein 3*), among others (Table 2 and Additional file 13: Table S5). On the other hand, interactions within the cell cycle, signaling, or cytoskeleton organization subnetwork were mostly from experimental evidence (Fig. 6a). In the case of this subnetwork, genes such as *CSLD5* (*Cellulose synthase-like D5*), *TUB1* (*Tubulin beta-1 chain*), *TUA2* (*Tubulin alpha-2 chain*), *TUA4* (*Tubulin alpha-4 chain*), *CYCB1; 4* (*G2/mitotic-specific cyclin-B*), *CDKB2;2* (*Cyclin-dependent kinase B2–2*), and *POK2* (*Phragmoplast orienting kinesin 2*), among others, were found (Table 2 and Additional file 13: Table S5). Finally, an independent network formed by transcription factors was mainly involved in the circadian rhythm (Phytoclock 1, PCL1; Pseudo-response regulator 5, PRR5; Early flowering 4, ELF4) and auxin responses (Auxin response factor 4, ARF4; Auxin-responsive proteins IAA29 and IAA30) (Fig. 6a).

**Table 2:** List of representative Arabidopsis orthologs of PS DEGs (nodes) forming subnetworks as shown in Fig. 6

DEG	Cluster	***P. vulgaris*** ID	Arabidopsis ortholog gene	Gene symbol	Function
**Up-regulated**	**Cell wall dynamics**	Phvul.009G242700	*AT5G44030*	*CESA4*	Cellulose synthase A4; required for beta-1,4-glucan microfibril crystallization, a major mechanism of the cell wall formation
		Phvul.009G090100	*AT4G18780*	*IRX1*	IRREGULAR XYLEM 1; required for beta-1,4-glucan microfibril crystallization, a major mechanism of the cell wall formation
		Phvul.003G154600	*AT5G17420*	*IRX3*	IRREGULAR XYLEM 3; required for beta-1,4-glucan microfibril crystallization, a major mechanism of the cell wall formation
		Phvul.008G029000	*AT5G15630*	*IRX6*	IRREGULAR XYLEM 6, a COBRA-like extracellular glycosyl-phosphatidyl inositol-anchored protein family involved in secondary cell wall biosynthesis
		Phvul.006G065800	*AT2G38080*	*IRX12*	Laccase-4; required for secondary xylem cell wall lignification
		Phvul.009G148800	*AT3G18660*	*PGSIP1*	Plant glycogenin-like starch initiation protein 1; glycosyltransferase required for the addition of both glucuronic acid and 4-O-methylglucuronic acid branches to xylan in stem cell walls
		Phvul.001G021800	*AT4G33330*	*PGSIP3*	Plant glycogenin-like starch initiation protein 3; glycosyltransferase required for the addition of both glucuronic acid and 4-O-methylglucuronic acid branches to xylan in stem cell walls
		Phvul.005G091200	*AT5G54690*	*GAUT12*	Galacturonosyltransferase 12; involved in pectin assembly and/or distribution, and in the synthesis of secondary wall glucuronoxylan
		Phvul.007G026900	*AT1G68560*	*XYL1*	Alpha-xylosidase 1; glycoside hydrolase releasing xylosyl residues from xyloglucan oligosaccharides at the non-reducing end
		Phvul.006G133700	*AT5G49720*	*GH9A1*	Endoglucanase 25; required for cellulose microfibrils formation. Involved in cell wall assembly during cell elongation and cell plate maturation in cytokinesis
		Phvul.009G016100	*AT1G75680*	*GH9B7*	Endoglucanase 10, glycosyl hydrolase 9B7. Endohydrolysis of (1- > 4)-beta-D-glucosidic linkages in cellulose, lichenin and cereal beta-D-glucans
		Phvul.007G218400	*AT4G02290*	*GH9B13*	Endoglucanase 17, glycosyl hydrolase 9B13. Endohydrolysis of (1- > 4)-beta-D-glucosidic linkages in cellulose, lichenin and cereal beta-D-glucans
		Phvul.010G123100	*AT3G14310*	*PME3*	Pectinesterase 3; acts in the modification of cell walls via demethylesterification of cell wall pectin
		Phvul.008G288800	*AT4G12730*	*FLA2*	Fasciclin-like arabinogalactan 2; may be a cell surface adhesion protein
		Phvul.005G011900	*AT3G10720*	*AT3G10720*	Pectinesterase 25; acts in the modification of cell walls via demethylesterification of cell wall pectin
		Phvul.009G252200	*AT3G16850*	*AT3G16850*	Pectin lyase-like superfamily protein
		Phvul.006G028800	*AT4G23820*	*AT4G23820*	Pectin lyase-like superfamily protein
	**Cell cycle, signaling and cytoskeleton organization**	Phvul.001G211000	*AT1G02730*	*CSLD5*	Cellulose synthase like D5; 1,4-beta-D-xylan synthase involved in stem and root growth
		Phvul.009G017300	*AT1G75780*	*TUB1*	Tubulin beta; tubulin is the major constituent of microtubules
		Phvul.009G114100	*AT1G50010*	*TUA2*	Tubulin alpha-2 chain; tubulin is the major constituent of microtubules
		Phvul.007G047300	*AT1G04820*	*TUA4*	Tubulin alpha-4 chain. Encodes an alpha tubulin isoform, an structural constituent of cytoskeleton
		Phvul.008G203300	*AT2G26760*	*CYCB1;4*	Cyclin B1;4, a G2/mitotic-specific cyclin-B involved in centrosome formation and ciliogenesis
		Phvul.001G000500	*AT1G20930*	*CDKB2;2*	Cyclin-dependent kinase B2–2, regulation of G2/M transition of mitotic cell cycle
		Phvul.003G293500	*AT3G19050*	*POK2*	Phragmoplast orienting kinesin 2; involved in the spatial control of cytokinesis by a proper phragmoplast guidance
		Phvul.007G159100	*AT2G37420*	*AT2G37420*	ATP binding microtubule motor family protein; responsible for microtubule translocation
		Phvul.006G052700	*AT3G20150*	*AT3G20150*	Kinesin motor family protein, ATP-dependent microtubule motor activity
		Phvul.002G093500	*AT1G70950*	*TPX2*	Targeting protein for Xklp2; microtubule-associated protein (MAP) that regulates the orientation of interphase cortical microtubules
		Phvul.001G028200	*AT1G10200*	*WLIM1*	LIM domain-containing protein WLIM1; binds to actin filaments and promotes cross-linking into thick bundles
		Phvul.009G082500	*AT2G26330*	*TE1*	ERECTA; receptor kinase that, together with ERL1 and ERL2, regulates aerial architecture, including inflorescence and stomatal patterning
		Phvul.007G063200	*AT5G62230*	*ERL1*	ERECTA-like 1; receptor kinase that regulates inflorescence architecture and organ shape as well as stomatal patterning, including density and clustering, together with ER and ERL2
		Phvul.002G196200	*AT5G46330*	*MPL12.8*	FLAGELLIN-SENSITIVE 2; constitutes the pattern-recognition receptor (PPR) that determines the specific perception of flagellin (flg22)
		Phvul.008G017400	*AT3G28040*	*AT3G28040*	Leucine-rich receptor-like protein kinase family protein, probably inactive
		Phvul.005G099100	*AT5G45970*	*RAC2*	RAC-like 2; inactive GDP-bound Rho GTPases reside in the cytosol, are found in a complex with Rho GDP-dissociation inhibitors
		Phvul.006G115500	*AT1G01200*	*RABA3*	Rab family protein; intracellular vesicle trafficking and protein transport
**Down-regulated**	**Protein folding**	Phvul.004G107700	*AT5G52640*	*HSP90.1*	Heat shock protein 81–1; functions as a holding molecular chaperone which stabilizes unfolding protein intermediates
		Phvul.004G162100	*AT3G24500*	*MBF1C*	Multiprotein bridging factor 1C; involved in the tolerance to heat and osmotic stress
		Phvul.004G044100	*AT1G74310*	*HSP101*	Heat shock protein 101; molecular chaperone that plays an important role in thermotolerance
		Phvul.009G078300	*AT2G26150*	*HSFA2*	Heat shock transcription factor A2; transcriptional activator involved in heat stress responses
		Phvul.011G065000	*AT3G12580*	*HSP70*	Heat shock protein 70; a coactivator involved in the regulated transcription of nearly all RNA polymerase II-dependent genes
		Phvul.003G154800	*AT1G16030*	*Hsp70b*	Heat shock protein 70B; in cooperation with other chaperones, stabilize preexistent proteins against aggregation and mediate the folding of newly translated polypeptides
		Phvul.008G013000	*AT5G02500*	*HSC70–1*	Heat shock 70 kDa protein 1/8; a coactivator involved in the regulated transcription of nearly all RNA polymerase II-dependent genes
		Phvul.008G095600	*AT3G08970*	*ATERDJ3A*	DnaJ domain-containing protein; regulates protein folding in the endoplasmic reticulum (ER) lumen
		Phvul.010G024500	*AT3G25230*	*ROF1*	Rotamase FKBP 1; co-chaperone that positively modulates thermotolerance by interacting with HSP90 and increasing the HSFA2-mediated accumulation of chaperones of the small-HSPs family
		Phvul.009G046500	*AT4G27670*	*HSP21*	Heat shock protein 21; protein processing in endoplasmic reticulum
		Phvul.011G016100	*AT4G25200*	*HSP23.6*	Small heat shock protein 23.6; protein processing in endoplasmic reticulum
		Phvul.010G024500	*AT3G25230*	*ROF1*	Rotamase FKBP 1; co-chaperone that positively modulates thermotolerance by interacting with HSP90 and increasing the HSFA2-mediated accumulation of chaperones of the small-HSPs family
		Phvul.010G155300	*AT1G52560*	*AT1G52560*	HSP20-like chaperone; protein processing in endoplasmic reticulum
		Phvul.002G095400	*AT1G23100*	*AT1G23100*	GroES-like protein; chaperone cofactor-dependent protein refolding
	**Chloroplast-associated processes**	Phvul.009G259600	*AT2G46830*	*CCA1*	Protein CCA1; transcription factor involved in the circadian clock and in the phytochrome regulation
		Phvul.008G022800	*AT3G02380*	*COL2*	CONSTANS-like 2; putative transcription factor involved in chloroplast organization
		Phvul.001G061400	*AT5G24120*	*SIGE*	Sigma factor E; essential for blue light-mediated transcription of psbD, which encodes the photosystem II reaction center protein D2
		Phvul.010G018200	*AT3G17609*	*HYH*	HY5-homolog; transcription factor that promotes photomorphogenesis in light
		Phvul.007G226300	*AT5G03555*	*NCS1*	Nucleobase cation symporter 1; nucleobase-proton symporter that facilitates uracil import into plastids
		Phvul.008G254400	*AT3G21150*	*BBX32*	B-box 32 protein; repressor of light-mediated regulation of seedling development
		Phvul.006G040800	*AT4G27030*	*FADA*	Fatty acid desaturase A; fatty acid desaturase involved in the production of chloroplast-specific phosphatidylglycerol molecular species
		Phvul.005G113200	*AT3G21890*	*BBX31*	B-box domain protein 31; involved in the CO-mediated long-day flowering-promotion pathway

Concerning to down-regulated genes, 102 Arabidopsis orthologs out of 223 interact with each other (Fig. 6b). Two subnetworks protruded from the main network, the first one being associated with protein folding processes, whereas the second was composed of genes associated with chloroplast processes (Fig. 6b). Importantly, interactions within the first subnetwork were mostly supported by experimental data (purple lines). Specifically, genes involved in protein folding, such as *HSP90.1* (*Heat shock protein 81–1*), *MBF1C* (*Multiprotein bridging factor 1c*), *HSP101* (*Heat shock protein 101*), *HSFA2* (*Heat shock transcription factor A2*), *HSP70* (*Heat shock protein 70*), *HSP70B* (*Heat shock protein 70B*), *HSC70–1* (*Heat shock 70 KDa protein 1/8*), *ATERDJ3A* (*DnaJ domain-containing protein*), *ROF1* (*Rotamase FKBP 1*), *HSP21* (*Heat shock protein 21*), *HSP23.6* (*Small heat shock protein 23.6*), *AT1G52560* (*HSP20-like chaperone*), and *AT1G23100* (*GROES-like protein*) were found forming this subnetwork (Table 2 and Additional file 13: Table S5). The second subnetwork was composed of genes such as *CCA1* (*Protein CCA1*), *COL2* (*Constans-like 2*), *SIGE* (*Sigma factor E*), *HYH* (*HY5-homolog*), *NCS1* (*Nucleobase cation symporter 1*), *BBX32* (*B-box 32 protein*), *FADA* (*Fatty acid desaturase A*), and *BBX31* (*B-box domain protein 31*). Such components are associated with chloroplast processes, mainly responses to light and abiotic stimuli (Table 2 and Additional file 13: Table S5). Altogether, the functional protein association networks for a subset of DEGs with orthologs in Arabidopsis indicate that drought stress causes, the up-regulation of genes associated with plant cell wall dynamics, among other processes, and repression of genes that participate in protein folding and chloroplast processes in *P. vulgaris* PS drought-tolerant cultivar.

## Discussion

Since scarce molecular data are available regarding drought tolerance for those varieties of *P. vulgaris* with drought tolerance features such as the PS cultivar, here we have assessed its transcriptional profile during drought stress. Firstly, phenotypic and physiological changes after drought treatment of PS, AH, and NP cultivars showed differences in their response, which are in agreement with their genetic variability among the tested common bean plants [16, 35, 41, 45, 48]. The phenotypic inspection, in combination with the assessment of a physiological parameter such as PSII efficiency during drought and recovery, showed that PS is more tolerant to drought than AH and NP (Figs. 1 and 2). As reduced photosynthetic rate during drought is mainly the consequence of stomatal closure, the better recovery observed in PS, might be the result of a controlled balance between effective stomatal closure regulation and conservation of tissue hydration to sustain plant growth during drought stress [47, 49–52]. Such a scenario could explain the observation of PS behavior during drought stress, namely its major biomass of aerial tissues as reflected by the comparison of FW and DW values (Additional file 3: Fig. S3a). Indeed, a recent report has found that drought tolerance of PS is in part, by maintaining a high photosynthesis rate under limited water supply [50]. Interestingly, the drought tolerance of PS in our greenhouse conditions agrees with previous studies showing the same trait under field conditions [35, 47, 53]. However, a remarkable difference with those preceding reports is that this study was carried out at an early stage of plant development, indicating that PS is tolerant to drought even at earlier stages of development, which represents an advantage for plant development under such stressing conditions.

On the other hand, common bean plants utilize diverse mechanisms to cope with drought, such as tissue water retention, osmotic adjustment, integrity of membrane system, and stomata adjustment [47, 49, 54–57]. Since the RNA-Seq technology allows to explore relevant correlations and construct models to describe biological states [58, 59], the assessment of PS transcriptome using this technology allowed us to detect global transcriptional variations between control and drought-treated plants of the PS cultivar at earlier stages of its vegetative development if compared to previous studies. Overall, most of the up-regulated genes in PS in response to drought belong to processes related to plant cell wall re-modeling and polysaccharide metabolic processes, whereas repressed genes are associated with protein folding, chloroplast (mainly responses to light and abiotic stimuli), and oxidation-reduction processes (Fig. 4 and Fig. 5) (see also Additional file 8: Table S2 and Additional file 11: Fig. S8). Accordingly, the prediction of subcellular localization supports the importance of an increase of extracellular proteins during drought response of PS, together with the reduction of cytoplasmic, chloroplastic and mitochondrial proteins percentages (Fig. 4c and d). The interpretation of DEGs found in drought-treated plants is more complicated than anticipated, especially for those DEGs being down-regulated. However, an analysis of GO terms, functional classification, and interactions among DEGs helped to formulate some hypotheses.

Drought stress affects plant cell wall integrity thus giving rise to complex and dynamic behavior, involving either its loosening or tightening to maintain growth [60–63]. In general, cell wall-related genes identified in PS were mostly involved in secondary cell wall (SCW) dynamics (Fig. 6, Table 2, and Additional file 13: Table S5). SCWs are produced by specialized plant cell types and are particularly important in those cells to provide mechanical support. In brief, SCWs are composed of cellulose, hemicellulose, and lignin, as well as cell wall-associated proteins [64]. The cellulose synthase complex (CSC) carries out the synthesis of cellulose intended for SCWs and basically is formed by CesA4, CesA7, and CesA8 proteins (also known as IRX5, IRX3, and IRX1, respectively) [65]. Interestingly, all core components of CSC were found among the up-regulated genes in PS, forming a subnetwork identifiable within the main network (Fig. 6a and c). Intriguingly, although mutations encoding the CSC (*cesA4*, *cesA7*, and *cesA8)* show defects in secondary cell wall formation, the *cesA8* mutant has increased tolerance to drought and osmotic stress [66]. Thus, the up-regulation of *CESA8* (and *CESA4* and *CESA7*) in PS highlights the complexity of drought-derived responses, which probably depend on the plant species and/or tissue-specific and temporal expression of such genes. None withstanding, the discovery in *A. thaliana* that photosynthetic activity is a major regulator of cellulose synthesis and deposition [67] could suggest that drought tolerance of PS is given by its sustained PSII efficiency under limited water supply, thereby maintaining its growth. This is supported by the major biomass of aerial tissues observed when FW and DW values were compared (Additional file 3: Fig. S3a). In that sense, the finding that genes involved in plant cell wall re-modeling are up-regulated in PS (as well as in AH and NP in the case of *XTH6* and *CESA4* genes, as shown in Additional file 7: Fig. S6) by drought stress is in agreement with several studies. For instance, a drought-tolerant common bean known as PHB-0683 has been found to change the expression of the cell wall or extracellular proteins in response to water-stress, suggesting that drought caused important changes in the cell wall structure in the common bean plant [68]. Another drought-tolerant common bean variety, known as BAT 477, has also been analyzed at the transcriptional level under drought conditions [69]. Among other terms, Pereira and collaborators identified the metabolism of polysaccharides as one of the processes that highlight during drought response. Also, overexpression of thaumatin-like protein genes (*TLPs*) involved in the SCW development has been shown to enhance drought tolerance in tobacco plants [70, 71].

Other up-regulated genes involved in cell wall re-modeling like xyloglucan-modifying enzymes, endoglucanases, arabinogalactan proteins, pectinesterases, pectin lyase-like proteins, among others, also formed a subnetwork (Fig. 6a and Table 2). Interactions among these cell wall re-modeling proteins are supported by co-expression (Fig. 6a), thus, they could play a role in drought tolerance according to previous findings [72, 73]. Indeed, *PGSIP1* and *PGSIP3* (two enzymes involved in xylan modification) have also been found up-regulated by drought, suggesting that SCW strength contributes to common bean tolerance [74]. Also, overexpression of a xyloglucan modifying enzyme (*XTH*) gene from *Capsicum annuum* (*CaXTH3*) in Arabidopsis and tomato resulted in seedlings showing an increased drought and salt tolerance [75, 76]. Consistent with those results, genes coding for cell wall degrading enzymes have been found down-regulated in black poplar drought-tolerant genotypes; and highly induced in drought-sensitive genotypes, resulting in cell wall loosening and leave senescence [77]. Also, over-expression of a pectin methylesterase inhibitor protein gene (*PMEI*), which inhibits extracellular pectinolytic enzymes that degrade cell wall pectin polymers, results in enhanced drought tolerance in Arabidopsis [78]. In addition to these evidences, a rice mutant in a glycophosphatidylinositol-anchored membrane protein encoded by *CLD/SRL1* gene is affected in SCW formation and has reduced drought tolerance [79]. Altogether with numerous additional studies in different plant species, drought tolerance seems to be related to an increase in cellulose and xyloglucan synthesis, as well as lignification [62, 73, 80–83]. However, other studies have found down-regulation of several cell wall-related genes, or increased cell wall elasticity parameters in response to water stress [84–89], suggesting that cell wall is dynamically restructured in a developmental stage-, tissue-, intensity-, and time-dependent manner to reach the shown traits on drought response in different plant species and varieties.

Several studies have shown that the cell wall not only plays a structural role but also senses and transmits stress signals to the interior of the cell [61, 62]. Surprisingly, a subnetwork formed by membrane-associated proteins, transmembrane receptor-like kinases, signaling factors, cell cycle regulators, components involved in cytoskeleton reorganization, and phragmoplast formation, is supported by experimental evidence (Fig. 6a and Table 2). For instance, a member of the Cellulose Synthase Like-D family, known as *CSLD5*, is an important node within the subnetwork (and was also up-regulated in AH as shown in Additional file 7: Fig. S6). Among the five CSLDs in Arabidopsis, only *CSLD5* is expressed predominantly in aerial organs [90]. Moreover, *csdl5* plants are hypersensitive to osmotic stress imposed by water deficit in the soil [91], supporting its putative role in drought tolerance in common bean. Although not completely clear, it seems that *CSLD5* or *CSLD5*-dependent cell wall components have a critical role in osmotic stress tolerance, likely involving the regulation of reactive oxygen species [91]. It has been hypothesized that cellulose synthase-like proteins, being part of the cell wall-membrane-cytoskeleton continuum, could be important for turgor sensing [92]. In addition, cell wall perturbations caused by abiotic stress likely involve members of different receptor-like kinases (RLKs). RLKs comprise a very large family of integral plasma membrane proteins and are believed to perceive changes in the extracellular space environment [93–96]. Interestingly, *Erecta* (*ER*) and *Erecta-like 1* (*ERL1*), the best-characterized genes affecting drought- and thermo-tolerance features [97–100], were found within the subnetwork of up-regulated genes (Fig. 6a and Table 2). On the other hand, *Flagellin-sensitive 2* (*AT5G46330*) and another *Leucine-rich receptor-like protein kinase* (*LRR-RLK*) (*AT3G28040*) were also found as important nodes within the subnetwork (Fig. 6a and Table 2). The absence of literature associating these RLKs with drought stress in plants, combined with the finding of their up-regulation in PS, deserve efforts to unravel their roles, if any, in drought tolerance. Albeit the encoded RLK by *AT3G28040* is predicted to be catalytically inactive, a recent finding indicates its physical interaction with the membrane-associated transcription factor *ANAC089* [101]. Again, the role of this inactive RLK, as well as its partner (*ANAC089*), merit more research regarding their putative roles in drought stress responses. Lastly, a GTPase known as *ROP7*/*ARAC2* protruded from the subnetwork (Fig. 6a and Table 2). Since the intracellular kinase domain of LRR-RLK proteins transduces the signal to kinase cascades when activated by Rop/Rac GTPases, some of the LRR-RLKs found within the network could likely be responsible for signal perception and transduction of cell wall-derived cues under drought stress. Notably, a study focused on xylan biosynthesis found that *ROP7*/*ARAC2* is one of the conserved components for SCW biosynthesis in both Arabidopsis and rice [102]. Indeed, *ROP7*/*ARAC2* is expressed specifically during the late stages of xylem differentiation in Arabidopsis [103], suggesting that it is a key regulator during SCW development and can be crucial for the signaling perception. Altogether, drought stress seems to trigger dedicated signaling pathways analogous to the fungal cell-wall integrity pathway, deserving more research in the future to unravel their specific roles in drought tolerance.

Finally, to those DEGs found down-regulated in PS, the group of genes involved in protein folding formed the most important subnetwork (Fig. 6b and Table 2). Since plant heat shock proteins (HSPs) facilitate protein folding or assembly under diverse developmental and adverse environmental conditions [104–109], many studies have shown that their overexpression can improve the tolerance of transgenic plants to drought and heat [110–114]. Moreover, the expression of HSPs under stress has been cataloged as intense, rapid, and transient, likely because plants are in an emergency response to the drought stress [115–117]. This could explain why we found down-regulated HSPs after 2 weeks of drought in PS since their function should be at the beginning of drought stress. Interestingly, one of the HSP coding genes showed no or limited down-regulation on AH and NP, respectively (Additional file 7: Fig. S6). In summary, the finding that HSPs are down-regulated in PS after 2 weeks of drought treatment suggests that these proteins are not required at this point but at the beginning of the stress response. This rapid and transient behavior can also be applicable to oxidation/reduction processes found in the group of down-regulated genes since the so-called ‘oxidative burst’ triggered by stress occur in this way. In addition, ‘oxidative burst’ has effects not only at the transcriptional level but post-transcriptional regulation levels are also involved in a time-dependent manner [118]. Altogether, the knowledge derived from this work is critical for the understanding of molecular mechanisms involved in drought tolerance, especially for an important crop such as common bean.

## Conclusions

In México, there are common bean cultivars capable of withstanding stress conditions by water deficit. These drought-tolerant cultivars represent ideal systems to study common bean tolerance to drought stress, and to use these gene sources to improve common bean varieties that are more sensitive to drought. In this study, we compared some physiological traits among three common bean cultivars that have been successfully cultivated in semiarid lands in the north of México (PS, AH, and NP), especially the PS, which is a drought-tolerant cultivar. This encouraged the identification of key DEGs in this cultivar after drought stress treatment in an early stage of plant development. In general, most of the up-regulated genes were involved in plant cell-wall dynamics and polysaccharide metabolic processes, whereas down-regulated genes were associated with protein folding, chloroplast, and oxidation-reduction processes. Our findings suggest that SCW properties contribute to *P. vulgaris* L. drought tolerance through alleviation or mitigation of drought-induced osmotic disturbances, making drought-tolerant cultivars more adaptable to such stress. Unraveling the complex mechanisms of drought tolerance is challenging and requires more intensive and integrative studies to find key functional components or molecular machinery that can be used as tools for engineering and breeding drought-tolerant crops. For instance, biotechnological tools aimed to increase cell wall properties and integrity could improve resilience to a changing climate in the future.

## Methods

### Plant material and growth conditions

Three well-known genotypes of the common bean, PS, AH and NP, were used in this study. PS and NP belong to the Mesoamerican gene pool, whereas AH is from the Andean gene pool [22]. Certified seeds corresponding to PS (FRI-040-251,104) and AH (747-FRI-001-220,995) were obtained from the National Institute for Forest and Agricultural Research (INIFAP). In the case of NP (AP78/Mo-91-92–2029-20 M genotype), such cultivar was kindly provided by INIFAP-Campo Experimental del Valle del Fuerte, México [119]. Seeds were soaked in 96% ethanol for 1 min. Then, ethanol was discarded, and 50% sodium hypochlorite was added for 5 to 12 min, depending on the cultivar (5 min for AH, 8 min for PS and 12 min for NP) with constant agitation. Finally, seeds were washed five times with sterile distilled water before planting in sterile aluminum trays containing a layer of wet sheets of paper. Trays were covered with aluminum foil and incubated at 30 °C for a week. Then, seedlings were transferred into plant pots containing sterile vermiculite as substrate and grown under greenhouse conditions. All plants were watered with Hoagland’s basal salt solution in increasing concentrations every week (from 0.1X to 1X) to fulfill increasing growth demands. For the experiments, plants at the development stage V4, showing three fully expanded trifoliate leaves (45-days after planting), were randomized and subjected to the described water regimes. Each experimental unit was composed of twenty-four pots with two plants per pot, considering eight replicates. Accordingly, plants without any treatment (Control), drought-treated plants (Drought), and post-drought recovery plants (Recovery) were established. Whereas plants of the control group (C) grew under continuous irrigation, drought-treated plants (D) were subjected to a period of progressive water deficit for 2 weeks by suppression of irrigation. The drought treatment was stopped when plants showed clear symptoms of stress like small leaves, dark green foliage color, leaf wilting and folding, leaf drop, as well as premature senescence. A group of plants subjected to the drought treatment (60-days old plants after transplanting) was re-watered with Hoagland’s solution to allow plant recovery for 2 weeks and classified as post-drought recovery plants (R) (74-days after transplanting). During the experiment, phenotypic (photographic) record and physiological measurements were taken at indicated times. Finally, at the end of the experiments, aboveground plant tissues of PS cultivar (including all trifoliates, petioles, internodes, and stems, and excluding senescent primary leaves) were sampled and pooled, followed by quick-freezing with liquid nitrogen and stored at − 80 °C for further analysis. All samples from all experiments were harvested between 9:00 and 10:00 h considering circadian and temperature effects. At least two biologically independent experiments were performed for this study, and plant materials from six to nine plants were pooled for each group.

### Physiological measurements

Plant growth was measured and expressed as relative growth (RG). RG values considered the plant height (from the substrate surface to the apical tip of main stem) at the beginning of the drought treatment as 1. In the case of photosystem II (PSII) efficiency, the maximum Quantum Yield (QY) was determined using a fluorometer (intensity of the saturation pulse equals approximately 3000 μmol.m^− 2^.s^− 1^ and lasts ca. 1 s.) (Fluorpen FP 100, PSI Instruments, Czech Republic). QY measurements were always taken on the upper adaxial right side of the leaves tip avoiding the midrib. Measurements were made on primary leaves and the central folio from all expanded trifoliates, of at least six to nine light-pre-adapted plants (equivalent to *F*_*v*_*’/F*_*m*_*′*, ratio of variable to maximum fluorescence of open PSII) from each experimental condition on each independent experiment. For fresh plant weight determination, roots and shoots were sampled followed by weight measurement with a precision balance (Voyager®, Ohaus Corporation, Parsippany, USA). Then, the same samples were dried for a week at 70 °C in a DHG-9145A Hinotek oven, and dry weight was measured. One-way ANOVA was used to compare the statistical difference between measurements (*P* < 0.05). Graphs indicate mean with a 95% confidence interval. Shown data are representative from at least two independent experiments.

### RNA extraction

The previously pooled and frozen aerial plant samples were powdered by grinding the frozen tissue in liquid nitrogen. Each pool included 6–9 plants of each cultivar under control conditions or drought treatment. Thus, 12 RNA samples were extracted in two replicates under either drought or control conditions. Total RNA was extracted using about 45 mg of the powdered sample and added with 700 μL of the Z6-extraction buffer (8 M guanidinium-HCl, 20 mM MES, 20 mM EDTA, 50 mM β-mercaptoethanol, pH 7.5). Then, an equal volume of phenol:chloroform:isoamyl alcohol (25:24:1) was added to carry out the extraction of RNA, followed by purification using the ssDNA/RNA Clean & Concentrator™ kit (Zymo Research Corp, Orange, CA, USA), according to the manufacturer’s instructions. Equal amounts of RNA from control or drought conditions samples were pooled together for further analysis, resulting in two RNA populations, one for control conditions and one for drought treatment. The RNA integrity was verified by agarose gel electrophoresis and the Agilent 2100 bioanalyzer (Agilent Technologies, Palo Alto, CA).

### RNA-Seq analysis

For assessing the transcriptome of PS under control and drought treatment, libraries were constructed corresponding to each condition using the TruSeq RNA Sample Preparation Kit (Illumina, Inc., San Diego, US-CA), following the manufacturer’s recommendations. In brief, poly(A)-tailed mRNA was enriched and fragmented, followed by first-strand cDNA synthesis. Subsequent second strand cDNA synthesis and the final reactions were cleaned up before performing the end repair step, and the addition of a single adenylate into the 3’ends. Adapters were ligated to both ends of the short fragments, which were enriched by 36 PCR cycles and validated. cDNA fragments pools were loaded to Illumina MiSeq (Illumina, Inc., San Diego, US-CA) platform for single-ended sequencing. Illumina reads (GSE123381) were trimmed and filtered using Trimmomatic [120], followed by quality-assessment using the FastQC tool (https://www.bioinformatics.babraham.ac.uk/projects/fastqc/). Low-quality reads were discarded, and the generated clean data were aligned to the *P. vulgaris* reference genome (G19833) [32] using TopHat [121]. The reference genome and gene annotation for *P. vulgaris L.* v2.1 were obtained from the Phytozome website (http://www.phytozome.net/). TopHat was run for alignment with mostly default settings, except for mismatches (−-read-mismatches 2) and intron length (−-min-intron-length 40, −-max-intron-length 2000). Further analysis was carried out with RNA-Seq analysis approaches using programs of the Tuxedo suite [121–123]. Particularly, PS transcriptomes under Control (C) and Drought (D) conditions were reconstructed by using Cufflinks with the default parameters. To generate comprehensive transcripts for subsequent gene expression analysis, the assembled transcriptomes were subsequently merged by using Cuffmerge.

### Identification of differentially expressed genes

Cuffdiff was used to compare the transcripts expression level, and to test the statistical significance between two conditions [123]. Genes were ranked according to normalized fragments per kilobase per million mapped reads (FPKM) to identify differentially expressed genes (DEGs). FPKM values were assigned to each gene by comparing the FPKM value under the drought treatment to that in the control condition. Genes that were up- or down-regulated were considered as DEGs if their *P*-value was ≤0.05 [122].

### Annotation and functional classification of DEGs

To identify Gene ontology terms significantly enriched within the group of DEGs, up- and down-regulated genes were subjected to analysis using the free online platform of AgriGO (http://bioinfo.cau.edu.cn/agriGO/) (FDR correction and Fisher’s exact test ≤0.1) [124]. In the case of prediction of protein subcellular localization, it was performed with the CELLO tool [125]. For further analysis of DEGs, a search of the corresponding gene orthologs in the Arabidopsis genome was carried out. Then, the subset of DEGs with orthologs in Arabidopsis was used to identify biological pathways significantly enriched using PANTHER (http://www.pantherdb.org/) [126]. Also, manual classification of those DEGs was performed according to cellular processes. Finally, the same subset of DEGs with orthologs in Arabidopsis was subjected to analysis using the String software [127] to construct an interaction network of DEGs.

### Validation of DEGs with RT-PCR

To validate RNA-Seq results, eight genes were selected from the list of DEGs and subjected to semi-quantitative RT-PCR analysis. Primer pairs were designed for *PYL4* (*Pyrabactin resistance 1-like 4*), *XTH6* (*Xyloglucan endotransglucosylase/hydrolase 6*), *CESA4* (*Cellulose synthase A4*), *CSLD5* (*Cellulose synthase like-D5*), *HSP70* (*Heat shock protein 70*), *HSFA2* (*Heat shock transcription factor A2*), *FTSH6* (*FTSH protease 6*) and *HYH* (*HY5-homolog*). Constitutive genes for this study were selected from our RNA-Seq data (*EIF5A*, *Elongation initiation factor 5A*) or previously reported gene references suitable for abiotic stress experiments (*SKIP16*, *SKP1/ASK-interacting protein 16*) [128] (Additional file 14: Table S6). The total RNA was reverse-transcribed using the RevertAid H Minus First-Strand cDNA Synthesis Kit (ThermoScientific, USA), followed by semi-quantitative RT-PCR analysis (28, 30, 32, 34 and 36 cycles) with at least two independent replicates. The data obtained from different PCR runs were analyzed with ImageJ 1.52a (https://imagej.nih.gov/ij/download.html) by quantifying gel bands density values for each DEG. Density values were normalized according to the *EIF5A* constitutive expression on each condition, obtaining the relative transcript abundance for the selected DEGs. One-way ANOVA was used to compare the statistical difference between measurements (*P* < 0.05). Graphs indicate mean with a 95% confidence interval.

## Supplementary information

**Additional file 1: Figure S1.** PSII efficiency of the first true leaves and trifoliates on three common bean cultivars in response to drought stress. **a** Photosystem II efficiency (*F*_*v*_*’/F*_*m*_*′*) of the first true leaves after two weeks of drought treatment. **b, c** and **d** *F*_*v*_*’/F*_*m*_*′* of trifoliates 1, 2, and 3. Pinto Saltillo (PS), Azufrado Higuera (AH), and Negro Jamapa Plus (NP). C, Control; D, Drought. Graphical representation of mean ± SE of six to nine individual plants from each experiment, out of at least two independent biological experiments. Different letters indicate significant differences compared to the control plants.

**Additional file 2: Figure S2.** Changes in RG and *F*_*v*_*’/F*_*m*_*′* values of three common bean cultivars submitted to drought and then recovery. **a** and **b.** RG and *F*_*v*_*’/F*_*m*_*′* values of bean cultivars according to times before (day 0) and after two weeks of drought stress (days 7 and 14), as well as after two weeks of re-hydration (day 28), respectively. Shown *F*_*v*_*’/F*_*m*_*′* values correspond to measurements carried out for all trifoliates of all experiments, which varied from at least three to eight in some cases. In each case, C, D and R correspond to Control, Drought and Recovery, respectively. Graphical representation of at least two independent biological experiments is shown. This figure is an extension of Fig. 2a and b. Pinto Saltillo (PS), Azufrado Higuera (AH), and Negro Jamapa Plus (NP).

**Additional file 3: Figure S3.** Relationship between FW and DW values of the aerial part on three common bean cultivars. FW (black dotted bars) and DW (white bars with diagonal stripes) values corresponding to the three bean varieties are shown after two weeks of drought stress **(a)** and after two weeks of recovery (**b**). Values for FW correspond to the left side, whereas DW is shown on the right side. Control samples exhibit a slight relationship of a ten-fold decrease with regard to FW and DW values. Significant differences (*P* < 0.05) compared to the control plants are indicated by different letters. Pinto Saltillo (PS), Azufrado Higuera (AH), and Negro Plus (NP). C, Control; D, Drought.

**Additional file 4: Figure S4.** Robustness of the PS transcriptome analysis. **a** Density plot of the expression level (log10 FPKM) distribution for all genes in Control and Drought conditions. **b** A scatter plot showing the gene expression values of genes under Control (x-axis) and Drought (y-axis) conditions. Each point represents the expression of a gene under both conditions evaluated. Both plots were generated by CummeRbund.

**Additional file 5: Table S1.** List of DEGs of PS in response to drought stress (Excel file)

**Additional file 6: Figure S5.** Validation of PS DEGs in response to drought from an independent experiment. Semi-quantitative RT-PCR of up-regulated (upper panel) and down-regulated (lower panel) genes are shown at 32 cycles. *PYL4, XHT6, CESA4,* and *CSLD5* correspond to up-regulated genes, whereas *HSP70, HSFA2, FTSH6,* and *HYH* are the down-regulated ones. *SKP16* was used as the constitutive control. C and D indicate Control and Drought. N (No cDNA) and –RT are controls used in the RT-PCR experiments. M indicates the molecular marker (DNA ladder).

**Additional file 7: Figure S6.** Expression levels of DEGs in in AH and NP cultivars. **a.** Selected DEGs according to the network in Fig. 6 are shown regarding their expression levels in AH and NP (boxed). Expression of the same set of genes in PS is presented in Fig. 3. b and c. Density analyses of PCR bands were determined by ImageJ software and normalized using the *EIF5A* constitutive internal control corresponding to each condition (a.u. - arbitrary units) in AH and NP, respectively. Graphical representation of mean ± SE of at least three independent replicates. One-way ANOVA was used to compare the statistical difference between measurements (*P* < 0.05). Samples tested for the same gene are indicated by lowercase letters. Significant differences compared to the control samples are indicated by different numbers. C and D indicate Control and Drought, respectively; M indicates the molecular marker (DNA ladder). (*CSLD5* was not detected in NP under the used PCR conditions).

**Additional file 8: Table S2.** Gene ontology terms enriched among DEGs of PS under drought stress (pdf)

**Additional file 9: Figure S7.** Gene ontology terms enriched among DEGs with orthologs in Arabidopsis. GO terms enriched or depleted among the up- and down-regulated genes with orthologs in Arabidopsis (425 and 223, respectively) are shown. Classification is according to Biological process (BP), Molecular function (MF), or Cellular compartment (CC).

**Additional file 10: Table S3.** Gene ontology terms enriched among Arabidopsis orthologs of PS DEGs in response to drought stress (pdf)

**Additional file 11: Figure S8.** Enriched biological pathways in the Arabidopsis orthologs of PS DEGs in response to drought stress. Statistically over- or under-enriched biological pathways in the input list of DEGs (Pie charts in the right) are compared to the reference list of the total number of *Arabidopsis thaliana* genes (Pie charts in the left) using Fisher’s exact test. **a** PANTHER pie chart (right) of the over-represented biological pathways within the up-regulated genes. **b** Pie chart (right) corresponding to the down-regulated genes showing the under-represented biological pathways in this group of DEGs.

**Additional file 12: Table S4.** GENEMANIA and DAVID functional annotation (Excel file)

**Additional file 13: Table S5.** Classification of Arabidopsis orthologs of PS DEGs in response to drought stress according to cellular processes (Excel file)

**Additional file 14: Table S6.** Oligonucleotides used in this study (pdf)

## Data Availability

The datasets generated and analyzed during the current study are available in the NCBI-GEO (Gene Expression Omnibus) repository, GSE123381 (https://www.ncbi.nlm.nih.gov/geo/query/acc.cgi?acc=GSE123381).

## Abbreviations

PS: Pinto saltillo
AH: Azufrado higuera
NP: Negro jamapa plus
DEGs: Differentially expressed genes
SCW: Secondary cell wall
ASERCA: Apoyos y servicios a la comercialización agropecuaria
RG: Relative growth
QY: Quantum yield
FW: Fresh weight
DW: Dry weight
RNA-Seq: RNA-sequencing
FPKM: Fragments per kilobase per million
GO: Gene ontology
SEA: Singular enrichment analysis
CSC: Cellulose synthase complex
C: Control irrigated plants
D: Drought-treated plants
R: Post-drought recovery plants
NCBI: National centre for biotechnology information
GEO: Gene expression omnibus
