# Supplementary figures and images for: Genome-wide transcriptional changes triggered by water deficit on a drought-tolerant common bean cultivar
Source: BMC Plant Biol. 2020 Nov 17;20:525. doi: 10.1186/s12870-020-02664-1 (PMC7672829; doi:10.1186/s12870-020-02664-1)

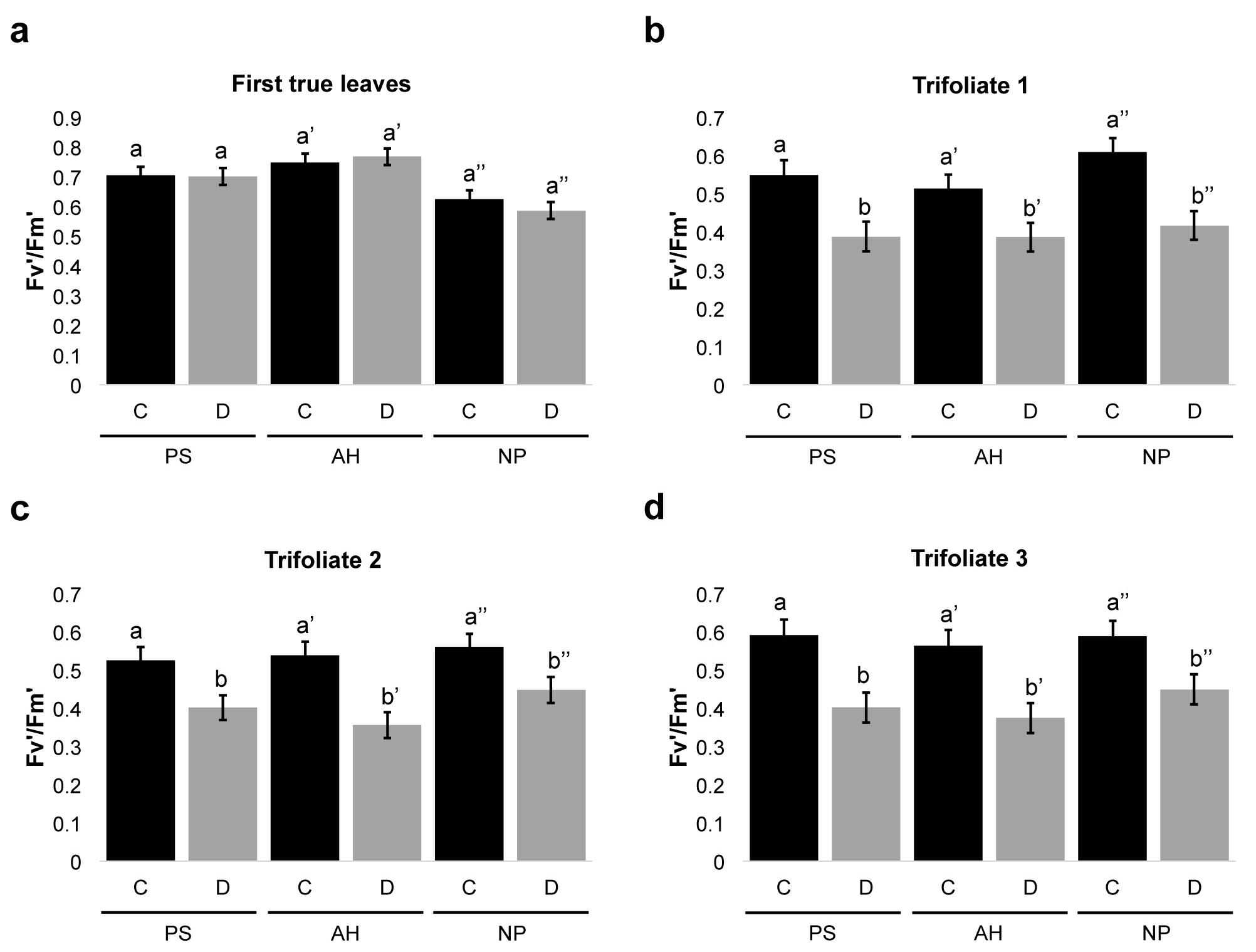

Supplement: Supplementary file 1 — Additional file 1: Figure S1. PSII efficiency of the first true leaves and trifoliates on three common bean cultivars in response to drought stress. a Photosystem II efficiency (Fv’/Fm′) of the first true leaves after two weeks of drought treatment. b, c and d Fv’/Fm′ of trifoliates 1, 2, and 3. Pinto Saltillo (PS), Azufrado Higuera (AH), and Negro Jamapa Plus (NP). C, Control; D, Drought. Graphical representation of mean ± SE of six to nine individual plants from each experiment, out of at least two independent biological experiments. Different letters indicate significant differences compared to the control plants. [file 12870_2020_2664_MOESM1_ESM.tif]

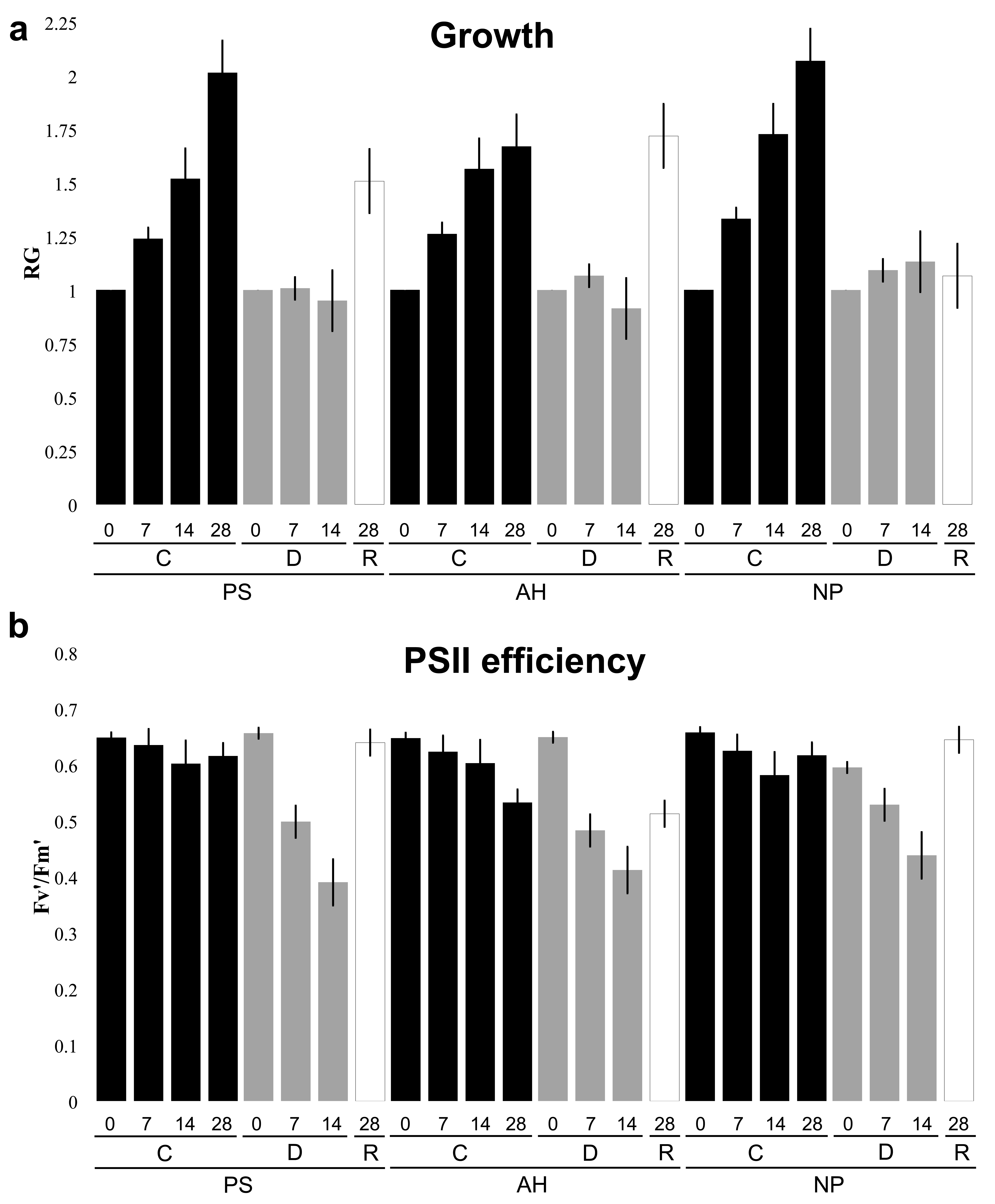

Supplement: Supplementary file 2 — Additional file 2: Figure S2. Changes in RG and Fv’/Fm′ values of three common bean cultivars submitted to drought and then recovery. a and b. RG and Fv’/Fm′ values of bean cultivars according to times before (day 0) and after two weeks of drought stress (days 7 and 14), as well as after two weeks of re-hydration (day 28), respectively. Shown Fv’/Fm′ values correspond to measurements carried out for all trifoliates of all experiments, which varied from at least three to eight in some cases. In each case, C, D and R correspond to Control, Drought and Recovery, respectively. Graphical representation of at least two independent biological experiments is shown. This figure is an extension of Fig. 2a and b. Pinto Saltillo (PS), Azufrado Higuera (AH), and Negro Jamapa Plus (NP). [file 12870_2020_2664_MOESM2_ESM.jpg]

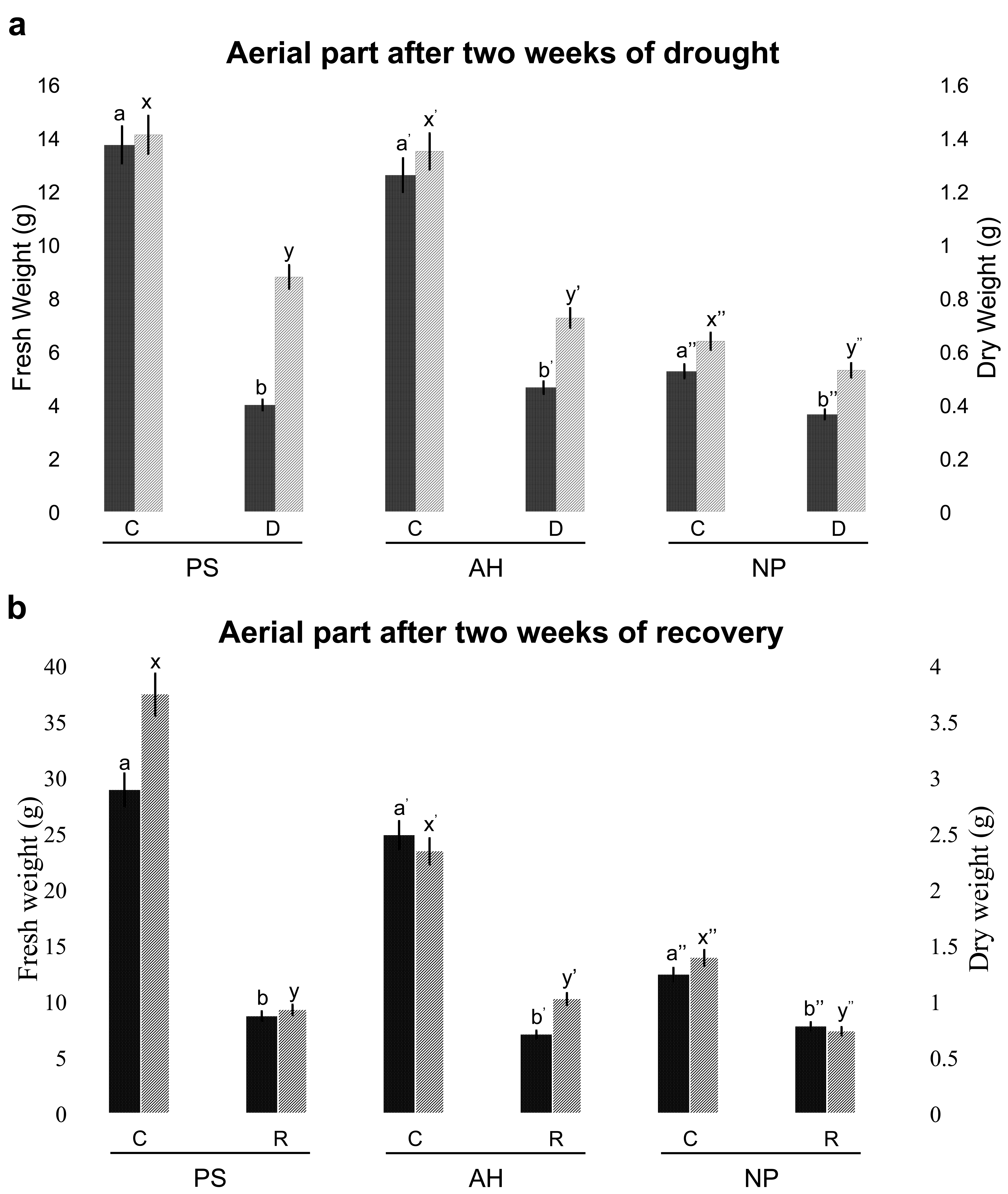

Supplement: Supplementary file 3 — Additional file 3: Figure S3. Relationship between FW and DW values of the aerial part on three common bean cultivars. FW (black dotted bars) and DW (white bars with diagonal stripes) values corresponding to the three bean varieties are shown after two weeks of drought stress (a) and after two weeks of recovery (b). Values for FW correspond to the left side, whereas DW is shown on the right side. Control samples exhibit a slight relationship of a ten-fold decrease with regard to FW and DW values. Significant differences (P < 0.05) compared to the control plants are indicated by different letters. Pinto Saltillo (PS), Azufrado Higuera (AH), and Negro Plus (NP). C, Control; D, Drought. [file 12870_2020_2664_MOESM3_ESM.jpg]

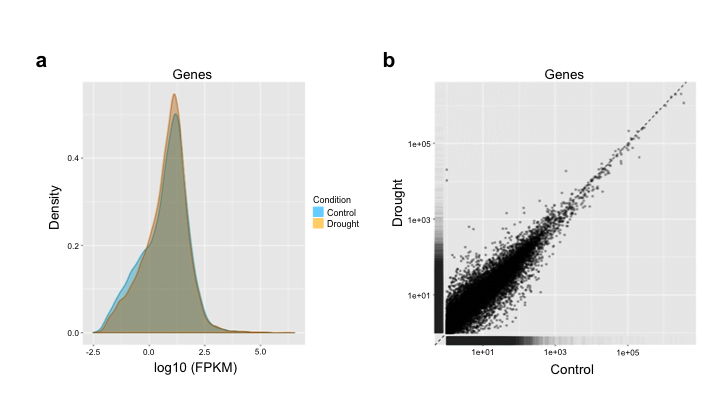

Supplement: Supplementary file 4 — Additional file 4: Figure S4. Robustness of the PS transcriptome analysis. a Density plot of the expression level (log10 FPKM) distribution for all genes in Control and Drought conditions. b A scatter plot showing the gene expression values of genes under Control (x-axis) and Drought (y-axis) conditions. Each point represents the expression of a gene under both conditions evaluated. Both plots were generated by CummeRbund. [file 12870_2020_2664_MOESM4_ESM.tiff]

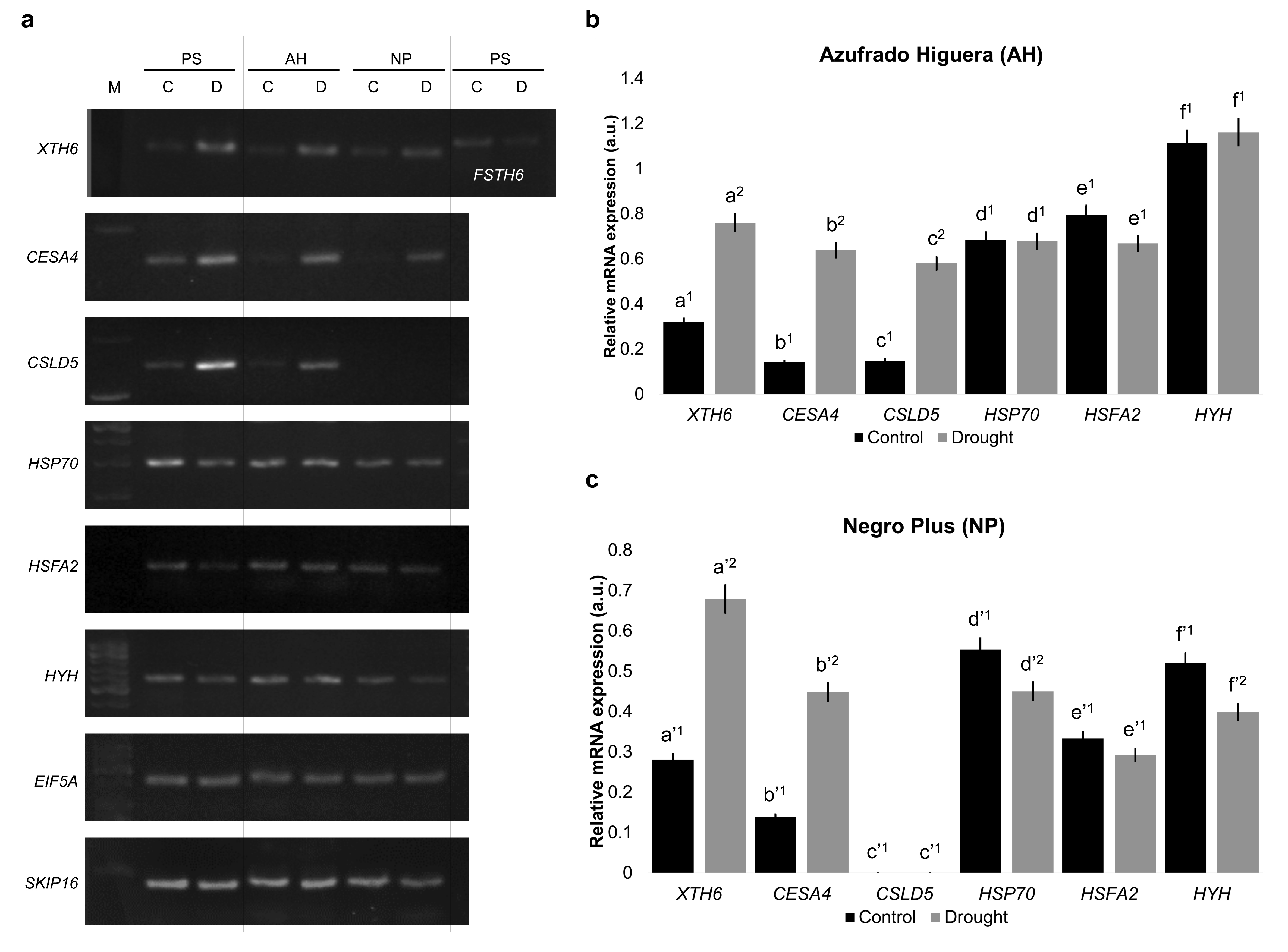

Supplement: Supplementary file 7 — Additional file 7: Figure S6. Expression levels of DEGs in in AH and NP cultivars. a. Selected DEGs according to the network in Fig. 6 are shown regarding their expression levels in AH and NP (boxed). Expression of the same set of genes in PS is presented in Fig. 3. b and c. Density analyses of PCR bands were determined by ImageJ software and normalized using the EIF5A constitutive internal control corresponding to each condition (a.u. - arbitrary units) in AH and NP, respectively. Graphical representation of mean ± SE of at least three independent replicates. One-way ANOVA was used to compare the statistical difference between measurements (P < 0.05). Samples tested for the same gene are indicated by lowercase letters. Significant differences compared to the control samples are indicated by different numbers. C and D indicate Control and Drought, respectively; M indicates the molecular marker (DNA ladder). (CSLD5 was not detected in NP under the used PCR conditions). [file 12870_2020_2664_MOESM7_ESM.jpg]

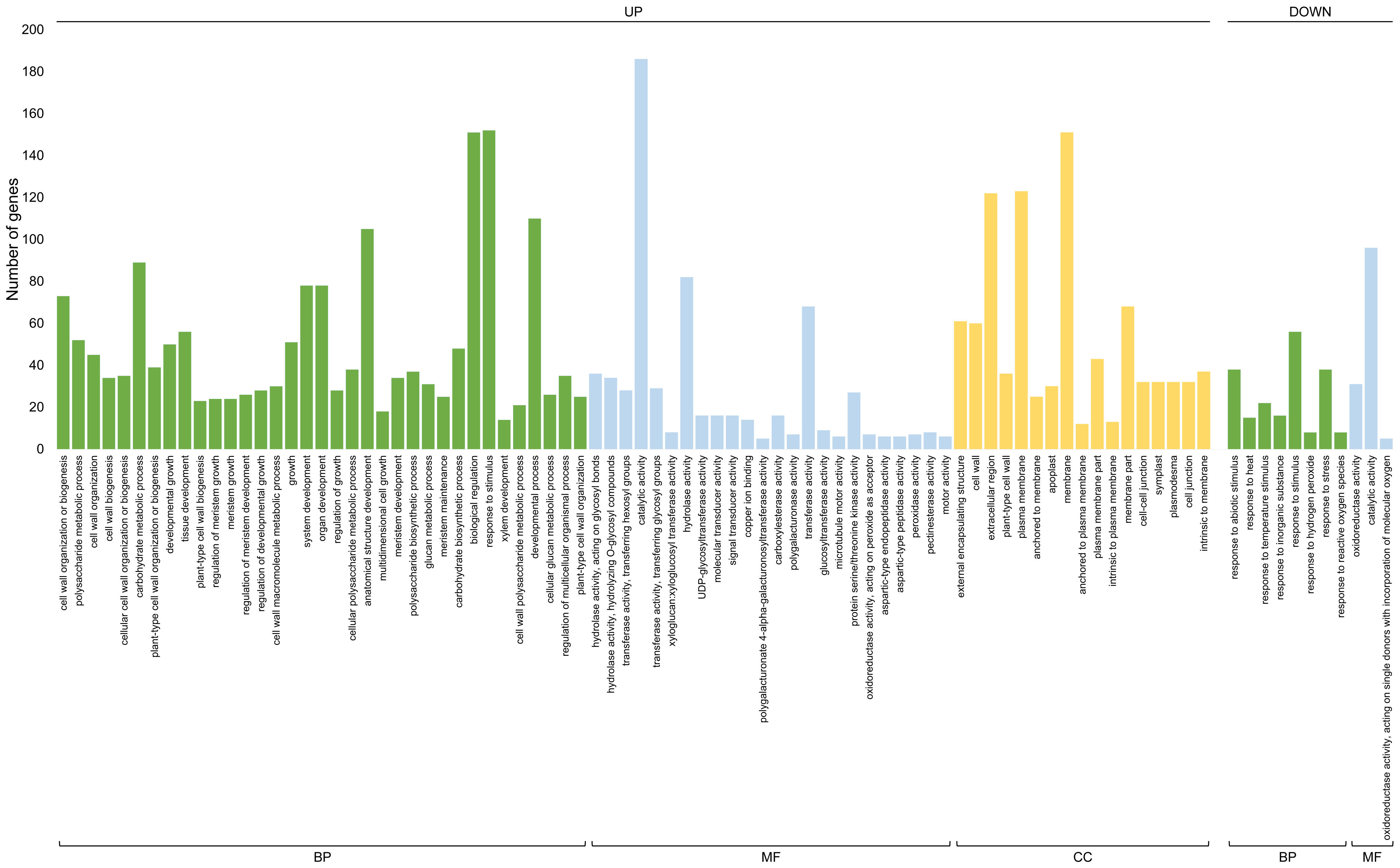

Supplement: Supplementary file 9 — Additional file 9: Figure S7. Gene ontology terms enriched among DEGs with orthologs in Arabidopsis. GO terms enriched or depleted among the up- and down-regulated genes with orthologs in Arabidopsis (425 and 223, respectively) are shown. Classification is according to Biological process (BP), Molecular function (MF), or Cellular compartment (CC). [file 12870_2020_2664_MOESM9_ESM.jpg]
