# Supplementary material for: Genome-wide transcriptional changes triggered by water deficit on a drought-tolerant common bean cultivar
Source: BMC Plant Biol. 2020 Nov 17;20:525. doi: 10.1186/s12870-020-02664-1 (PMC7672829; doi:10.1186/s12870-020-02664-1)
Supplement: Supplementary file 8 — Additional file 8: Table S2. Gene ontology terms enriched among DEGs of PS under drought stress (pdf) [file 12870_2020_2664_MOESM8_ESM.pdf]

Additional file 8: Table S2 Gene ontology terms enriched among DEGs of PS under drought stress

DEG*	GO term	Description	Genes	p-value	FDR
Up-regulated (382)	GO:0005975 (BP)	carbohydrate metabolic process	58	4.30E-13	2.40E-10
	GO:0006073 (BP)	cellular glucan metabolic process	18	1.60E-10	3.40E-08
	GO:0044042 (BP)	glucan metabolic process	18	1.80E-10	3.40E-08
	GO:0044092 (BP)	negative regulation of molecular function	14	2.60E-09	2.90E-07
	GO:0043086 (BP)	negative regulation of catalytic activity	14	2.60E-09	2.90E-07
	GO:0044264 (BP)	cellular polysaccharide metabolic process	19	6.20E-08	5.70E-06
	GO:0044238 (BP)	primary metabolic process	194	3.00E-07	2.30E-05
	GO:0005976 (BP)	polysaccharide metabolic process	19	4.60E-07	3.10E-05
	GO:0008152 (BP)	metabolic process	245	6.80E-06	0.00041
	GO:0050790 (BP)	regulation of catalytic activity	14	1.10E-05	0.00061
	GO:0065009 (BP)	regulation of molecular function	14	1.30E-05	0.00065
	GO:0044262 (BP)	cellular carbohydrate metabolic process	24	4.70E-05	0.0021
	GO:0006508 (BP)	Proteolysis	27	0.00022	0.0091
	GO:0030243 (BP)	cellulose metabolic process	7	0.00027	0.0091
	GO:0030244 (BP)	cellulose biosynthetic process	7	0.00024	0.0091
	GO:0071555 (BP)	cell wall organization	9	0.00027	0.0091
	GO:0007017 (BP)	microtubule-based process	10	0.00028	0.0091
	GO:0009250 (BP)	glucan biosynthetic process	7	0.0012	0.038
	GO:0016798 (MF)	hydrolase activity, acting on glycosyl bonds	44	6.60E-15	1.40E-12
	GO:0004553 (MF)	hydrolase activity, hydrolyzing O-glycosyl compounds	43	7.10E-15	1.40E-12
	GO:0016787 (MF)	hydrolase activity	109	6.80E-11	9.10E-09
	GO:0005507 (MF)	copper ion binding	19	7.70E-10	7.80E-08
	GO:0016762 (MF)	xyloglucan:xyloglucosyl transferase activity	11	4.00E-09	3.20E-07
	GO:0042802 (MF)	identical protein binding	14	5.30E-09	3.60E-07
	GO:0004252 (MF)	serine-type endopeptidase activity	15	3.70E-07	2.20E-05
	GO:0004175 (MF)	endopeptidase activity	23	3.00E-06	0.00015
	GO:0016757 (MF)	transferase activity, transferring glycosyl groups	30	4.40E-06	0.00019
	GO:0017171 (MF)	serine hydrolase activity	17	8.30E-06	0.00031
	GO:0008236 (MF)	serine-type peptidase activity	17	8.30E-06	0.00031
	GO:0070011 (MF)	peptidase activity, acting on L-amino acid peptides	28	1.10E-05	0.00036
	GO:0003824 (MF)	catalytic activity	230	1.20E-05	0.00037
	GO:0008233 (MF)	peptidase activity	28	2.60E-05	0.00074
	GO:0016760 (MF)	cellulose synthase (UDP-forming) activity	7	0.00012	0.0031
	GO:0016759 (MF)	cellulose synthase activity	7	0.00012	0.0031
	GO:0016758 (MF)	transferase activity, transferring hexosyl groups	22	0.0004	0.0096
	GO:0046527 (MF)	glucosyltransferase activity	7	0.0011	0.024
	GO:0035251 (MF)	UDP-glucosyltransferase activity	7	0.0011	0.024
	GO:0004857 (MF)	enzyme inhibitor activity	11	0.0014	0.027
	GO:0048046 (CC)	Apoplast	11	4.00E-09	2.00E-07

	GO:0005618 (CC)	cell wall	18	2.50E-09	2.00E-07
	GO:0030312 (CC)	external encapsulating structure	18	2.20E-08	7.50E-07
	GO:0005576 (CC)	extracellular region	11	4.70E-06	0.00012
	GO:0015630 (CC)	microtubule cytoskeleton	5	0.0021	0.042
Down-regulated (228)	GO:0055114 (BP)	oxidation reduction	47	1.20E-07	4.70E-05
	GO:0032502 (BP)	developmental process	6	6.50E-05	0.013
	GO:0006633 (BP)	fatty acid biosynthetic process	6	0.00033	0.044
	GO:0016491 (MF)	oxidoreductase activity	52	8.90E-08	2.20E-05
	GO:0005506 (MF)	iron ion binding	20	2.60E-05	0.0033
	GO:0016746 (MF)	transferase activity, transferring acyl groups	13	0.00023	0.019
	GO:0016747 (MF)	transferase activity, transferring acyl groups other than amino-acyl groups	12	0.00041	0.026

*DEG = Differentially expressed gene; GO = Gene ontology; BP = Biological Process; MF = Molecular Function; CC = Cellular Component; FDR = false discovery ratio
