# Supplementary material for: Genome-wide transcriptional changes triggered by water deficit on a drought-tolerant common bean cultivar
Source: BMC Plant Biol. 2020 Nov 17;20:525. doi: 10.1186/s12870-020-02664-1 (PMC7672829; doi:10.1186/s12870-020-02664-1)
Supplement: Supplementary file 10 — Additional file 10: Table S3. Gene ontology terms enriched among Arabidopsis orthologs of PS DEGs in response to drought stress (pdf) [file 12870_2020_2664_MOESM10_ESM.pdf]

Additional file 10: Table S3. Gene ontology terms enriched among Arabidopsis orthologs of PS DEGs in response to drought stress (docx)

DEG	Ontology	GO term	Description	Genes	p-value	FDR
Up-regulated (425)	Biological process (BP)	GO:0071554	cell wall organization or biogenesis	73	4.10E-29	1.60E-25
		GO:0005976	polysaccharide metabolic process	52	3.80E-20	7.20E-17
		GO:0071555	cell wall organization	45	1.30E-17	1.60E-14
		GO:0042546	cell wall biogenesis	34	2.20E-17	2.10E-14
		GO:0070882	cellular cell wall organization or biogenesis	35	4.00E-17	2.80E-14
		GO:0005975	carbohydrate metabolic process	89	5.80E-17	2.80E-14
		GO:0071669	plant-type cell wall organization or biogenesis	39	5.80E-17	2.80E-14
		GO:0048589	developmental growth	50	5.60E-17	2.80E-14
		GO:0009888	tissue development	56	1.90E-16	8.10E-14
		GO:0009832	plant-type cell wall biogenesis	23	8.90E-16	3.40E-13
		GO:0010075	regulation of meristem growth	24	1.90E-15	6.50E-13
		GO:0035266	meristem growth	24	4.70E-15	1.50E-12
		GO:0048509	regulation of meristem development	26	6.80E-15	2.00E-12
		GO:0048638	regulation of developmental growth	28	8.30E-15	2.30E-12
		GO:0044036	cell wall macromolecule metabolic process	30	1.10E-14	2.70E-12
		GO:0040007	growth	51	1.30E-14	3.10E-12
		GO:0048731	system development	78	1.20E-13	2.60E-11
		GO:0048513	organ development	78	1.20E-13	2.60E-11
		GO:0040008	regulation of growth	28	1.40E-13	2.70E-11
		GO:0044264	cellular polysaccharide metabolic process	38	3.80E-13	7.40E-11
		GO:0048856	anatomical structure development	105	4.70E-13	8.60E-11
		GO:0009825	multidimensional cell growth	18	7.20E-13	1.30E-10
		GO:0048507	meristem development	34	9.60E-13	1.60E-10
		GO:0000271	polysaccharide biosynthetic process	37	1.00E-12	1.60E-10
		GO:0044042	glucan metabolic process	31	3.00E-12	4.60E-10
		GO:0010073	meristem maintenance	25	8.20E-12	1.20E-09
		GO:0016051	carbohydrate biosynthetic process	48	3.70E-11	5.30E-09
		GO:0065007	biological regulation	151	1.20E-10	1.70E-08
		GO:0050896	response to stimulus	152	1.40E-10	1.90E-08

		GO:0010089	xylem development	14	1.90E-10	2.40E-08
		GO:0010383	cell wall polysaccharide metabolic process	21	2.00E-10	2.50E-08
		GO:0032502	developmental process	110	5.40E-10	6.50E-08
		GO:0006073	cellular glucan metabolic process	26	7.10E-10	8.20E-08
		GO:0051239	regulation of multicellular organismal process	35	1.10E-09	1.30E-07
		GO:0009664	plant-type cell wall organization	25	1.20E-09	1.30E-07
		GO:0008361	regulation of cell size	36	1.60E-09	1.70E-07
		GO:0009834	secondary cell wall biogenesis	10	1.70E-09	1.80E-07
		GO:0032535	regulation of cellular component size	36	2.40E-09	2.40E-07
		GO:0090066	regulation of anatomical structure size	36	2.40E-09	2.40E-07
		GO:0010087	phloem or xylem histogenesis	14	3.60E-09	3.50E-07
		GO:0010410	hemicellulose metabolic process	18	4.00E-09	3.80E-07
		GO:0010817	regulation of hormone levels	27	4.10E-09	3.80E-07
		GO:0050793	regulation of developmental process	38	4.30E-09	3.80E-07
		GO:0043481	anthocyanin accumulation in tissues in response to UV light	14	6.20E-09	4.70E-07
		GO:0043480	pigment accumulation in tissues	14	6.20E-09	4.70E-07
		GO:0010382	cellular cell wall macromolecule metabolic process	18	5.80E-09	4.70E-07
		GO:0043473	pigmentation	14	6.20E-09	4.70E-07
		GO:0043478	pigment accumulation in response to UV light	14	6.20E-09	4.70E-07
		GO:0043476	pigment accumulation	14	6.20E-09	4.70E-07
		GO:0043479	pigment accumulation in tissues in response to UV light	14	6.20E-09	4.70E-07
		GO:0009653	anatomical structure morphogenesis	60	6.40E-09	4.80E-07
		GO:0007275	multicellular organismal development	102	7.30E-09	5.40E-07
		GO:0045491	xylan metabolic process	17	1.00E-08	7.30E-07
		GO:0048767	root hair elongation	17	1.20E-08	8.30E-07
		GO:0016049	cell growth	33	2.70E-08	1.90E-06
		GO:0009826	unidimensional cell growth	27	2.80E-08	1.90E-06
		GO:0060560	developmental growth involved in morphogenesis	27	2.80E-08	1.90E-06
		GO:0007017	microtubule-based process	21	3.10E-08	2.00E-06

		GO:0010413	glucuronoxylan metabolic process	16	4.50E-08	2.90E-06
		GO:0045492	xylan biosynthetic process	16	4.90E-08	3.10E-06
		GO:0032501	multicellular organismal process	102	5.30E-08	3.30E-06
		GO:0070592	cell wall polysaccharide biosynthetic process	16	6.40E-08	3.80E-06
		GO:0044038	cell wall macromolecule biosynthetic process	16	6.40E-08	3.80E-06
		GO:0070589	cellular component macromolecule biosynthetic process	16	6.40E-08	3.80E-06
		GO:0065008	regulation of biological quality	54	1.00E-07	6.10E-06
		GO:0010015	root morphogenesis	24	1.20E-07	7.20E-06
		GO:0050789	regulation of biological process	122	1.80E-07	1.10E-05
		GO:0048588	developmental cell growth	23	4.80E-07	2.70E-05
		GO:0048364	root development	27	6.70E-07	3.70E-05
		GO:0022622	root system development	27	6.90E-07	3.80E-05
		GO:0033692	cellular polysaccharide biosynthetic process	25	7.40E-07	4.00E-05
		GO:0042221	response to chemical stimulus	96	1.40E-06	7.50E-05
		GO:0044085	cellular component biogenesis	47	1.60E-06	8.30E-05
		GO:0010054	trichoblast differentiation	19	1.80E-06	9.40E-05
		GO:0044238	primary metabolic process	223	2.00E-06	9.80E-05
		GO:0048765	root hair cell differentiation	18	1.90E-06	9.80E-05
		GO:0048764	trichoblast maturation	18	1.90E-06	9.80E-05
		GO:0044262	cellular carbohydrate metabolic process	53	2.00E-06	9.90E-05
		GO:0048469	cell maturation	18	2.00E-06	9.90E-05
		GO:0009411	response to UV	16	2.10E-06	0.0001
		GO:0009932	cell tip growth	19	2.20E-06	0.00011
		GO:0016126	sterol biosynthetic process	13	2.60E-06	0.00012
		GO:0006949	syncytium formation	6	2.80E-06	0.00013
		GO:0010053	root epidermal cell differentiation	19	2.80E-06	0.00013
		GO:0008152	metabolic process	251	2.90E-06	0.00013
		GO:0042545	cell wall modification	20	3.00E-06	0.00014
		GO:0021700	developmental maturation	18	3.20E-06	0.00014
		GO:0000902	cell morphogenesis	28	3.50E-06	0.00015
		GO:0009926	auxin polar transport	10	3.60E-06	0.00015
		GO:0016125	sterol metabolic process	13	4.00E-06	0.00017

		GO:0060918	auxin transport	10	5.40E-06	0.00023
		GO:0009914	hormone transport	10	5.90E-06	0.00024
		GO:0007167	enzyme linked receptor protein signaling pathway	11	8.20E-06	0.00033
		GO:0007169	transmembrane receptor protein tyrosine kinase signaling pathway	11	8.20E-06	0.00033
		GO:0023052	signaling	63	8.50E-06	0.00034
		GO:0009628	response to abiotic stimulus	68	8.50E-06	0.00034
		GO:0034637	cellular carbohydrate biosynthetic process	33	9.10E-06	0.00036
		GO:0048653	anther development	9	1.40E-05	0.00053
		GO:0032989	cellular component morphogenesis	28	1.40E-05	0.00056
		GO:0006084	acetyl-CoA metabolic process	10	1.60E-05	0.0006
		GO:0007018	microtubule-based movement	8	1.80E-05	0.00068
		GO:0007389	pattern specification process	17	3.10E-05	0.0011
		GO:0048468	cell development	26	3.20E-05	0.0012
		GO:0006694	steroid biosynthetic process	13	4.80E-05	0.0018
		GO:0007166	cell surface receptor linked signaling pathway	11	4.90E-05	0.0018
		GO:0010033	response to organic substance	67	6.00E-05	0.0022
		GO:0009416	response to light stimulus	36	7.10E-05	0.0025
		GO:0005982	starch metabolic process	13	7.10E-05	0.0025
		GO:0009987	cellular process	255	7.40E-05	0.0026
		GO:0042547	cell wall modification during multidimensional cell growth	5	0.00011	0.004
		GO:0048466	androecium development	11	0.00012	0.0042
		GO:0048443	stamen development	11	0.00012	0.0042
		GO:0009913	epidermal cell differentiation	19	0.00012	0.0042
		GO:0007010	cytoskeleton organization	20	0.00012	0.0042
		GO:0008544	epidermis development	19	0.00013	0.0043
		GO:0007398	ectoderm development	19	0.00013	0.0043
		GO:0009605	response to external stimulus	33	0.00014	0.0045
		GO:0030243	cellulose metabolic process	10	0.00014	0.0045
		GO:0008202	steroid metabolic process	13	0.00014	0.0045
		GO:0048869	cellular developmental process	40	0.0002	0.0065
		GO:0009314	response to radiation	36	0.00023	0.0072

		GO:0051707	response to other organism	39	0.00026	0.0082
		GO:0042446	hormone biosynthetic process	13	0.0004	0.013
		GO:0023034	intracellular signaling pathway	17	0.00041	0.013
		GO:0042219	cellular amino acid derivative catabolic process	9	0.00042	0.013
		GO:0023033	signaling pathway	19	0.00044	0.014
		GO:0009719	response to endogenous stimulus	42	0.00045	0.014
		GO:0000226	microtubule cytoskeleton organization	12	0.00048	0.014
		GO:0042402	cellular biogenic amine catabolic process	8	0.00054	0.016
		GO:0009725	response to hormone stimulus	37	0.00054	0.016
		GO:0010374	stomatal complex development	11	0.0006	0.018
		GO:0042743	hydrogen peroxide metabolic process	14	0.00074	0.021
		GO:0006833	water transport	9	0.00076	0.022
		GO:0042044	fluid transport	9	0.00076	0.022
		GO:0051704	multi-organism process	45	0.00079	0.022
		GO:0023060	signal transmission	44	0.00078	0.022
		GO:0023046	signaling process	44	0.00079	0.022
		GO:0009886	post-embryonic morphogenesis	18	0.0008	0.022
		GO:0050794	regulation of cellular process	94	0.00087	0.024
		GO:0006800	oxygen and reactive oxygen species metabolic process	14	0.001	0.028
		GO:0001666	response to hypoxia	7	0.001	0.029
		GO:0044283	small molecule biosynthetic process	45	0.0013	0.034
		GO:0070482	response to oxygen levels	7	0.0013	0.035
		GO:0006066	alcohol metabolic process	31	0.0013	0.036
		GO:0042445	hormone metabolic process	13	0.0016	0.042
		GO:0009607	response to biotic stimulus	41	0.0018	0.048
	Molecular function (MF)	GO:0016798	hydrolase activity, acting on glycosyl bonds	36	2.00E-15	9.00E-13
		GO:0004553	hydrolase activity, hydrolyzing O-glycosyl compounds	34	1.20E-14	2.80E-12
		GO:0016758	transferase activity, transferring hexosyl groups	28	4.80E-11	7.20E-09
		GO:0003824	catalytic activity	186	1.90E-08	2.10E-06
		GO:0016757	transferase activity, transferring	29	6.60E-08	5.90E-06

			glycosyl groups			
		GO:0016762	xyloglucan:xyloglucosyl transferase activity	8	1.30E-07	9.50E-06
		GO:0016787	hydrolase activity	82	3.00E-07	1.90E-05
		GO:0008194	UDP-glycosyltransferase activity	16	1.40E-06	7.80E-05
		GO:0060089	molecular transducer activity	16	1.30E-05	0.00056
		GO:0004871	signal transducer activity	16	1.30E-05	0.00056
		GO:0005507	copper ion binding	14	3.20E-05	0.0013
		GO:0047262	polygalacturonate 4-alpha-galacturonosyltransferase activity	5	7.10E-05	0.0026
		GO:0004091	carboxylesterase activity	16	0.00013	0.0045
		GO:0004650	polygalacturonase activity	7	0.00015	0.0049
		GO:0016740	transferase activity	68	0.00044	0.012
		GO:0046527	glucosyltransferase activity	9	0.00042	0.012
		GO:0003777	microtubule motor activity	6	0.00067	0.018
		GO:0004674	protein serine/threonine kinase activity	27	0.0012	0.03
		GO:0016684	oxidoreductase activity, acting on peroxide as acceptor	7	0.0019	0.041
		GO:0004190	aspartic-type endopeptidase activity	6	0.002	0.041
		GO:0070001	aspartic-type peptidase activity	6	0.002	0.041
		GO:0004601	peroxidase activity	7	0.0019	0.041
		GO:0030599	pectinesterase activity	8	0.0022	0.044
		GO:0003774	motor activity	6	0.0024	0.045
	Cellular component (CC)	GO:0030312	external encapsulating structure	61	6.80E-30	2.00E-27
		GO:0005618	cell wall	60	3.70E-29	5.40E-27
		GO:0005576	extracellular region	122	6.00E-27	5.80E-25
		GO:0009505	plant-type cell wall	36	1.00E-21	7.20E-20
		GO:0005886	plasma membrane	123	1.90E-17	1.10E-15
		GO:0031225	anchored to membrane	25	4.90E-13	2.40E-11
		GO:0048046	apoplast	30	3.50E-12	1.40E-10
		GO:0016020	membrane	151	3.60E-11	1.30E-09
		GO:0046658	anchored to plasma membrane	12	2.20E-09	6.80E-08
		GO:0044459	plasma membrane part	43	8.60E-09	2.50E-07
		GO:0031226	intrinsic to plasma membrane	13	1.00E-07	2.60E-06
		GO:0044425	membrane part	68	7.20E-07	1.70E-05
		GO:0005911	cell-cell junction	32	3.40E-06	6.60E-05

		GO:0055044	symplast	32	3.30E-06	6.60E-05
		GO:0009506	plasmodesma	32	3.30E-06	6.60E-05
		GO:0030054	cell junction	32	3.70E-06	6.60E-05
		GO:0031224	intrinsic to membrane	37	2.50E-05	0.00043
Down-regulated (223)	Biological process (BP)	GO:0009628	response to abiotic stimulus	38	2.60E-14	1.50E-11
		GO:0009408	response to heat	15	1.60E-13	4.60E-11
		GO:0009266	response to temperature stimulus	22	3.60E-13	6.80E-11
		GO:0010035	response to inorganic substance	16	2.50E-11	3.50E-09
		GO:0050896	response to stimulus	56	9.20E-10	1.00E-07
		GO:0042542	response to hydrogen peroxide	8	2.60E-09	2.50E-07
		GO:0006950	response to stress	38	1.10E-08	8.90E-07
		GO:0000302	response to reactive oxygen species	8	7.70E-08	5.50E-06
		GO:0009644	response to high light intensity	7	9.60E-08	6.10E-06
		GO:0009642	response to light intensity	8	1.20E-07	6.60E-06
		GO:0009416	response to light stimulus	17	1.40E-07	7.30E-06
		GO:0009314	response to radiation	17	2.10E-07	9.80E-06
		GO:0042221	response to chemical stimulus	33	2.50E-07	1.10E-05
		GO:0006979	response to oxidative stress	12	9.70E-07	3.90E-05
		GO:0006629	lipid metabolic process	17	1.30E-05	0.00049
		GO:0044255	cellular lipid metabolic process	14	2.30E-05	0.00082
		GO:0006633	fatty acid biosynthetic process	7	2.60E-05	0.00088
		GO:0032787	monocarboxylic acid metabolic process	11	3.90E-05	0.0012
		GO:0006631	fatty acid metabolic process	8	7.20E-05	0.0022
		GO:0010038	response to metal ion	8	0.0001	0.003
		GO:0008610	lipid biosynthetic process	10	0.00033	0.0089
		GO:0010033	response to organic substance	19	0.00042	0.011
		GO:0009737	response to abscisic acid stimulus	9	0.00048	0.012
		GO:0043436	oxoacid metabolic process	14	0.00065	0.014
		GO:0006082	organic acid metabolic process	14	0.00066	0.014
		GO:0019752	carboxylic acid metabolic process	14	0.00065	0.014
		GO:0046686	response to cadmium ion	6	0.00076	0.016
		GO:0042180	cellular ketone metabolic process	14	0.00083	0.017
		GO:0016053	organic acid biosynthetic process	9	0.00094	0.018
		GO:0046394	carboxylic acid biosynthetic process	9	0.00094	0.018
		GO:0006457	protein folding	7	0.0014	0.026
		GO:0009607	response to biotic stimulus	11	0.0016	0.028

	Molecular function (MF)	GO:0016491	oxidoreductase activity	31	9.40E-10	1.80E-07
		GO:0003824	catalytic activity	96	6.20E-09	5.80E-07
		GO:0016701	oxidoreductase activity, acting on single donors with incorporation of molecular oxygen	5	4.00E-06	0.00025
