# Supplementary material for: Genome-wide transcriptional changes triggered by water deficit on a drought-tolerant common bean cultivar
Source: BMC Plant Biol. 2020 Nov 17;20:525. doi: 10.1186/s12870-020-02664-1 (PMC7672829; doi:10.1186/s12870-020-02664-1)
Supplement: Supplementary file 14 — Additional file 14: Table S6. Oligonucleotides used in this study (pdf) [file 12870_2020_2664_MOESM14_ESM.pdf]

Additional file 14: Table S6 Oligonucleotides used in this study

Primer	Sequence	Product (bp)
PYL4-F	5’ AGAGCTGTCACGTCATTGGAG 3’	237
PYL4-R	5’ GTTGTTTCGTGGCGCTAAGT 3’	
XTH6-F	5’ TCTTGGGGAACCGTAGTGGT 3’	237
XTH6-R	5’ CTCCCATTGCCTGCATCCTT 3’	
CESA4-F	5’ TGACTCCGATGAGCATCAGG	408
CESA4-R	5’ TTGCCAGTAACACTTCCAGCTG 3’	
CSLD5-F	5’ GCTATTCTCAGCCCTTATAGGATGC 3’	416
CSLD5-R	5’ AATGTCAACAAAGCTCCACCATC 3’	
HSP70-F	5’ AATCAACACCGTTTTCGATGC 3’	329
HSP70-R	5’ TACGCATAACATTGAGCCCTGA 3’	
HSFA2-F	5’ AACACTCTTACACTCGCACCG 3’	363
HSFA2-R	ACCATAGGTGTTGAGCTGTCG 3’	
FTSH6-F	5’ GTGCAGACCTGGCAAACCTC 3’	217
FTSH6-R	5’ ACTGGATCATGTCCTGCGGT 3’	
HYH-F	5’ GTTTCTGCTCAACAAGCCCG 3’	172
HYH-R	5’ CGTCTACTTTTGGCCTTGCAT 3’	
EIF5A-F	5’ ACGAGGAACACCACTTCGAG 3’	171
EIF5A-R	5’ AGTGACACTTTGCGTGTCCA 3’	
SKIP16-F	5’ ATGTTGCCAGAGTCGCTTGT 3’	202
SKIP16-R	5’ ACACGCTTAACAAGAGGCCA 3’	
